# Customizing skills for assistive robotic manipulators, an inverse reinforcement learning approach with error-related potentials

**DOI:** 10.1038/s42003-021-02891-8

**Published:** 2021-12-16

**Authors:** Iason Batzianoulis, Fumiaki Iwane, Shupeng Wei, Carolina Gaspar Pinto Ramos Correia, Ricardo Chavarriaga, José del R. Millán, Aude Billard

**Affiliations:** 1 Learning Algorithms and Systems Laboratory (LASA), École Polytechnique Fédérale de Lausanne (EPFL), Lausanne, Switzerland; 2 Brain-Machine Interface (CNBI), École Polytechnique Fédérale de Lausanne (EPFL), Geneva, Switzerland; 3 Department of Electrical and Computer Engineering, University of Texas at Austin, Austin, TX USA; 4 Department of Neurology, University of Texas at Austin, Austin, TX USA

**Keywords:** Learning algorithms, Neural decoding, Translational research, Machine learning

## Abstract

Robotic assistance via motorized robotic arm manipulators can be of valuable assistance to individuals with upper-limb motor disabilities. Brain-computer interfaces (BCI) offer an intuitive means to control such assistive robotic manipulators. However, BCI performance may vary due to the non-stationary nature of the electroencephalogram (EEG) signals. It, hence, cannot be used safely for controlling tasks where errors may be detrimental to the user. Avoiding obstacles is one such task. As there exist many techniques to avoid obstacles in robotics, we propose to give the control to the robot to avoid obstacles and to leave to the user the choice of the robot behavior to do so a matter of personal preference as some users may be more daring while others more careful. We enable the users to train the robot controller to adapt its way to approach obstacles relying on BCI that detects error-related potentials (ErrP), indicative of the user’s error expectation of the robot’s current strategy to meet their preferences. Gaussian process-based inverse reinforcement learning, in combination with the ErrP-BCI, infers the user’s preference and updates the obstacle avoidance controller so as to generate personalized robot trajectories. We validate the approach in experiments with thirteen able-bodied subjects using a robotic arm that picks up, places and avoids real-life objects. Results show that the algorithm can learn user’s preference and adapt the robot behavior rapidly using less than five demonstrations not necessarily optimal.

## Abstract (web summary)

Teaching an assistive robotic manipulator to move objects in a cluttered table requires demonstrations from expert operators, but what if the experts are individuals with motor disabilities? Batzianoulis et al. propose a learning approach which combines robot autonomy and a brain-computer interfacing that decodes whether the generated trajectories meet the user’s criteria, and show how their system enables the robot to learn individual user’s preferred behaviors using less than five demonstrations that are not necessarily optimal.

## Introduction

Individuals with spinal cord injury (SCI) often experience permanent neurological deficits and severe motor disabilities, which impair their ability to perform even the simplest everyday tasks, such as reaching-to-grasp objects. Assistance from robots may enable patients to recover some of their lost dexterity by letting a robotic system to perform these task on their behalf. In those cases where residual muscular capabilities are not reliable enough, a way to control such assistive robotic system is through brain–computer interfaces (BCIs)^1,2^.

A BCI measures and decodes the subject’s neural activity, translating their motor intention into the corresponding actions of a robot arm. These BCIs decode cortical correlates of movement parameters such as velocity^3–5^ or position^6,7^, thus providing direct control of the robot arm. Nevertheless, even after rather long training sessions, BCI performance still suffers from large variability over time and is significantly slower than the corresponding human actions.

Moreover, operation of the system relies on a continuous modulation of the brain signals to control the robot’s motion. Such intense level of concentration may not be amenable to all users. Involuntary changes in the user’s mental state, as well as fatigue and workload, may deteriorate BCI performance^8,9^. Such continuous modulation of the brain signals is therefore meant to be imprecise and cannot be used reliably for tasks that require fast reactivity and high precision, such as when avoiding obstacles. Hence, to facilitate user’s learning and control, we propose to grant some authority to the robotic system, by developing a shared-control paradigm for obstacle avoidance that exploits a high-level cognitive brain signal, generic enough for different intended robot movements, combined with the ability of the robotic systems to plan and execute safe and efficient trajectories to reach the intended goals.

**Fig. 1:**
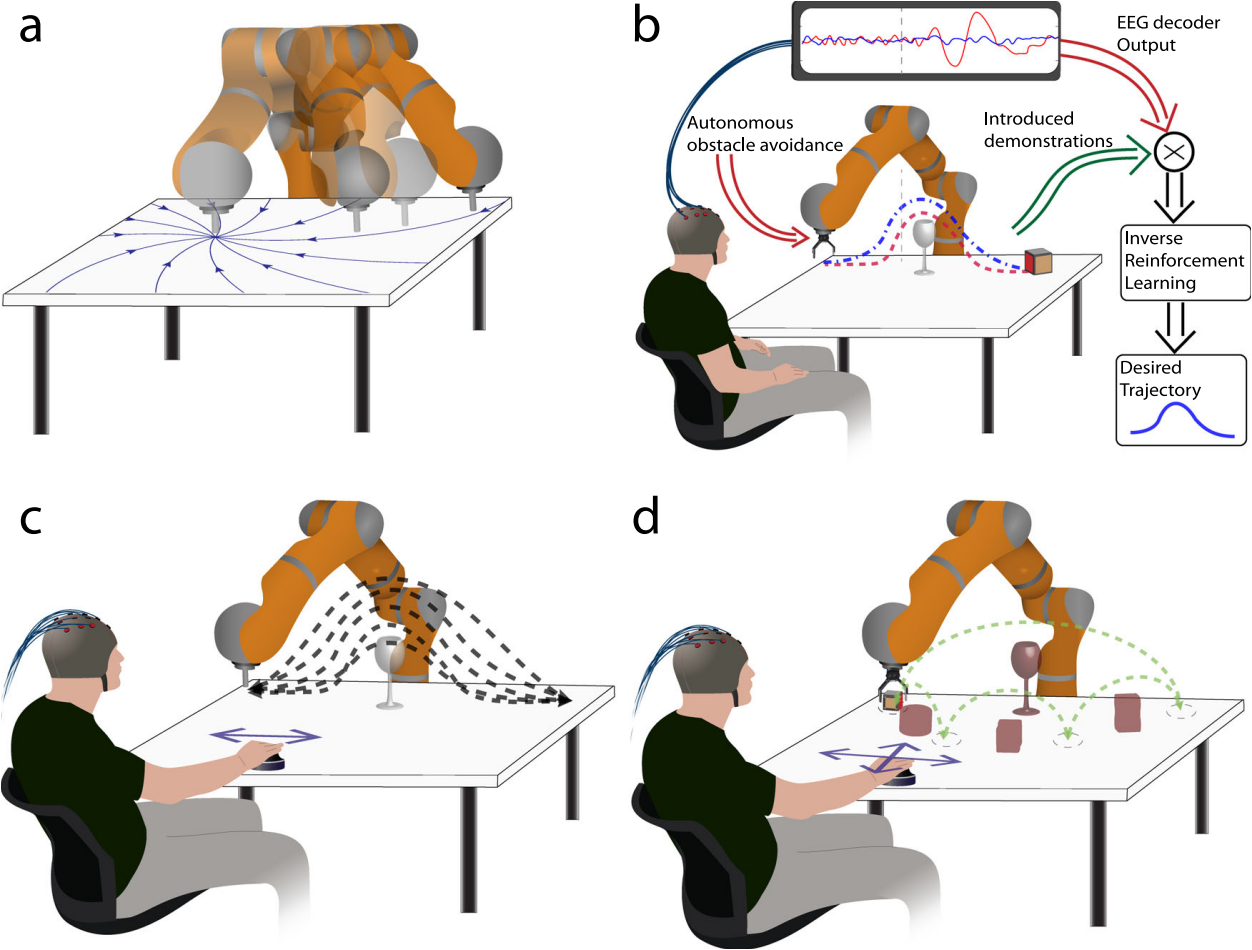
Overview of the control architecture and experimental protocol. **a** The robot follows trajectories generated from a planar dynamical system. The workspace of the robot (i.e., the table) is modeled with a vector-field and the robot’s trajectories are generated from the position initial position. Therefore, the robot follows a specific vector to reach its target. **b** An illustration of our approach. The robot moves towards the cube autonomously avoiding the glass with trajectories generated by a dynamical system. However, some trajectories (red dashed line) pass very close to the glass, creating a feeling of uncertainty to the user as the robot may collide with the glass (i.e., obstacle). This error expectation elicits ErrPs in the brain activity of the user and the output of the ErrPs decoder is associated with the robot trajectories. The desired trajectories are computed with the use of IRL. **c** The experimental protocol on the first experiment. The robot moves from left to right and vice versa performing an obstacle avoidance. The dashed dark lines correspond to the random trajectories of the robot, some of them could result in collision with the obstacle. The subject can deflect the joystick right or left to direct the robot accordingly or release the joystick for correcting the motion. This protocol corresponds to the calibration session of the second experiment too. **d** The experimental protocol in the second experiment. The subject commands the robot to grasp the object and place it on one of the four target positions (dashed circles) by pushing the joystick left, right, back or forward. The crimson objects correspond to the different obstacles placed in between the target positions. The green dashed line presents the target options for the user.

Motion planning has reached a high level of maturity for the control of robot arms^10^. A branch of motion planning is trajectory generation driven by dynamical systems (DS), where robot’s reaching motion toward a target is modeled through a vector field with one attractor located at the target (Fig. 1a). Once the target is defined, the robot’s velocity depends only on its position with respect to the target. The benefits of this method are, among others, that it enables real-time adaptation of the robot’s trajectory^11^. This makes it a natural framework for obstacle avoidance^12^, which exhibits high reactivity even in the presence of moving obstacles^13^.

Such a framework would avoid BCI depending solely on the user’s brain signals to drive the robotic arm, as the robot trajectories could be generated autonomously. However, these automatic trajectories might not be acceptable from the user’s perspective: the robot may approach the obstacle too closely or avoid it too sharply for their liking. We, therefore, propose a method that combines robot learning and BCI techniques by which the user can train the system to learn the obstacle-avoidance behaviors that suit her/his individual preferences.

A critical component of our approach is how to gather users’ preferences. Consider a situation where the default trajectories bring the robot very closely to the object (Fig. 1b). From where the user stands, this motion may appear as too risky or even leading to a collision. The user does not need to indicate such a perceived misbehavior explicitly, something that people suffering from severe motor disabilities can hardly do; it can be detected directly from the user’s error-related potentials (ErrP)^14,15^. Error-related potentials are time-locked brain potentials elicited when actions do not match users’ expectations^15–18^. ErrPs are employed in BCIs for correcting or adapting brain decoders^15,19,20^ and recently have been introduced for robot control^21–24^. While in previous works ErrPs were triggered by robot actions that are erroneous according to some explicit criteria, here we show that ErrPs are also elicited by error expectation—i.e., robot actions during its continuous movement that the user considers will lead to erroneous trajectories because they will not meet the user’s preferred obstacle avoidance behavior.

**Fig. 2:**
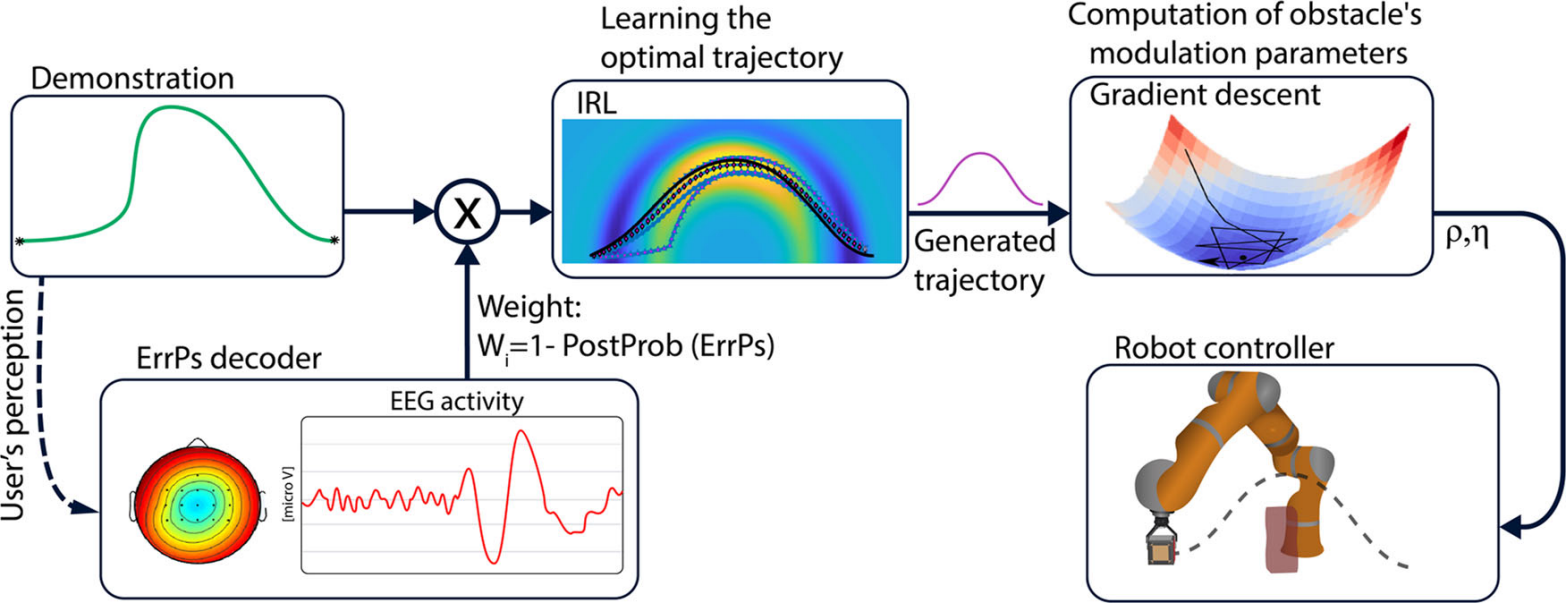
Information flow for training the robot’s controller. For each demonstration, the output of the ErrP decoder is converted into a weight and introduced to the IRL method together with the demonstration. Then, IRL infers a new trajectory that would lead to high reward on the basis of previous demonstrations. The resulting trajectory is afterward used to configure the DS-modulation parameters (*ρ*, *η*) using gradient descent. Finally, the controller uses these parameters to generate the next robot’s trajectory, based on a dynamical system approach, that should better reflect user’s preferences and guarantee obstacle avoidance.

In our framework, upon detecting the occurrence of ErrPs, the system adjusts its control policy to generate future trajectories that may better fit the user’s implicit reward function. For this purpose, we rely on inverse reinforcement learning (IRL), an approach that uses demonstrations from experts to both learn a reward function and to produce the optimal trajectory according to this reward^25–28^. IRL can hence be used in conjunction with ErrPs to determine when and how to update the intelligent robot controller, as illustrated in Fig. 2.

We validate this ErrP-IRL approach with 13 subjects in two series of experiments, as illustrated in Supplementary Fig. 1. In a first experiment, 8 subjects interacted with the robot arm via minimal commands delivered with a joystick to start the robot’s motion (deflecting the joystick toward the rough desired direction) and to signal error expectation (releasing the joystick). After the onset of the motion, the robot moved autonomously from left to right, or vice versa, using its dynamical system to avoid a wine glass sitting in the middle of the trajectory. We show that our IRL-ErrP approach derives the preferred trajectories for each subject. Then, in a second experiment, 5 additional participants used the same joystick to make the robot arm perform pick-and-place tasks, while avoiding obstacles, similar to daily tasks in a cluttered table. Picking and releasing an object was done by pressing the joystick button. Results not only show the feasibility of our approach and the rapid incremental learning of desired robot motion from a short number of demonstrations, but also that our approach enables the customization of robot trajectories according to the user’s individual preferences.

## Results

### Electrophysiological signature of ErrPs

**Fig. 3:**
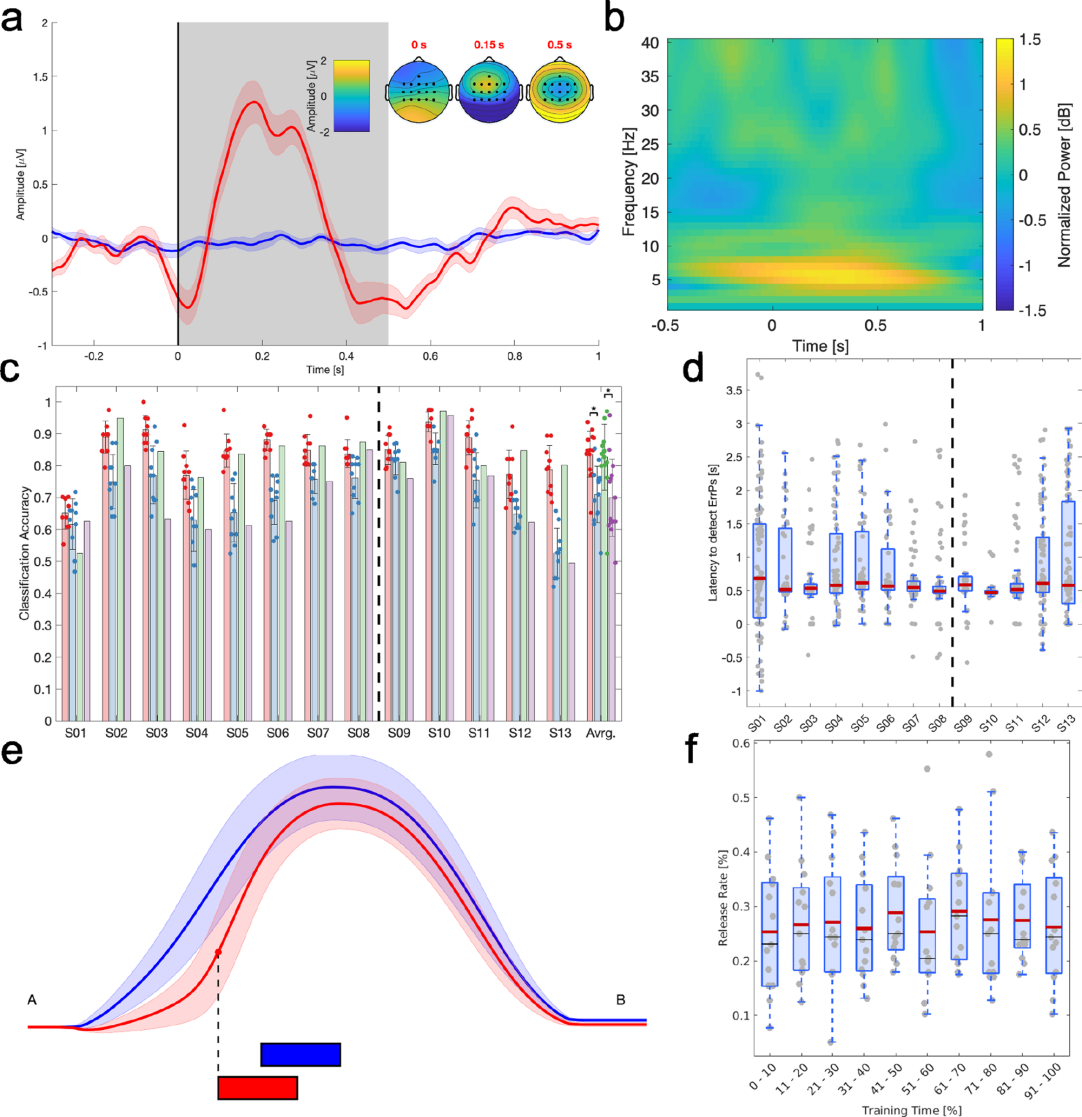
ErrP decoding results. **a** Grand average over all subjects (*N* = 13) of the EEG channel FCz, located in the fronto-central midline, for erroneous trajectories (*N* = 110 ± 32 per subject, in red) and for correct trajectories (*N* = 295 ± 25 per subject, in blue) during the calibration phase of the two experiments. For erroneous trials, time 0 s corresponds to the moment where subjects released the joystick; while for correct trials, the blue trace corresponds to the average EEG potential in the period [1.25, 2.5] s with respect to the onset of the robot motion. Gray area represents the time interval used for building the ErrP decoder. Inset: Topographical representation of EEG amplitude over the subjects scalp for erroneous trials at three different time points with respect to the onset; i.e., 0.00, 0.15, and 0.50 s. **b** Time-frequency analysis of the FCz channel, grand average over all erroneous trials and subjects. Time 0 s corresponds to the moment where subjects released the joystick. **c** Classification performance in four different conditions (mean ± std). The red, blue, green, and purple bars represent averaged time-locked classification accuracy of offline recordings, continuous classification accuracy of offline recordings, time-locked classification accuracy of online recordings, and continuous classification accuracy of online recordings, respectively. The vertical dotted line separates subjects who participated in the first experiment (S01-S08) from subjects who did in the second experiment (S09-S13). *p < 0.01. **d** Latency to detect ErrPs during continuous robot motion in the online phase (*N* = 73 ± 22 per subject). The box plot represents the distribution of the decoding latency with respect to the moment when subjects released the joystick (time 0 s). Vertical dotted line as in **c**. **e** Averaged correct and erroneous robot trajectories obtained during the calibration phase (mean ± std) of the two experiments. Black dashed line indicates the averaged release time, and the rectangles of each color indicate the time window used for computing the decoder (see section “Decoding the error-related potentials” for detail). **f** Error rate over 10% intervals of the calibration phase of the two experiments. Each dot corresponds to error rate of a subject during the specific time period of calibration, and the box plot illustrates the distribution of error rate for each interval of the calibration phase.

As hypothesized, error expectation (i.e., perception of an eventual collision) elicited ErrPs in subjects’ brain. Figure 3a and Supplementary Fig. 2 illustrate the grand average of all the data collected during the first and second experiments over all subjects (*N* = 13) of the EEG channel FCz, located in the fronto-central midline, for trajectories where the subject released the joystick to avoid a perceived collision (in red, *N* = 110 ± 32 per subject) and for trajectories where subjects did not feel the urge to stop the robot (in blue, *N* = 295 ± 25 per subject). This ErrP grand average has been obtained with a causal filter (4th order bandpass Butterworth filter with cut-off frequencies [1, 12] Hz) that is necessary for online real-time analysis of the EEG. Such a causal filter distorts the signal, what explains why the grand average does not resemble the usual waveform of an ErrP. Supplementary Fig. 3 reports the grand-averaged signals with the equivalent non-causal filtering (forward and backward, using the same Butterworth filter as for the causal version) that clearly exhibits the presence of the error-related negativity followed by a positive peak, which corresponds to the typical waveform of ErrPs although with different timing as reported in Fig. 3a. The reason for the appearance of the negative and positive peaks earlier than usual is that, in our case, EEG is synchronized to joystick release and not to the onset of the robot action that makes the subject judge the trajectory as risky. In line with previous works^15,21^, the sequential negative and positive deflections were observed for the erroneous condition. Also, as shown in the scalp-wide topographical representations (Fig. 3b, top-right and bottom-middle), the first positive peak at 0.15 s and the following negative at 0.5 s were strongly modulated over the fronto-central area.

A potential confounding factor that may give rise to the EEG potential associated with erroneous trials is that subjects released the joystick. Such a change in motor behavior should be accompanied by modulations of the mu and beta rhythms (broadly [8, 30] Hz) over the controlateral motor cortex that could extend over adjacent areas, the fronto-central midline FCz in particular. However, as depicted in Fig. 3b, the main modulations observed in FCz is an increase of the theta rhythm ([4, 8] Hz), which is considered as the main oscillatory pattern of error monitoring^15,17,29^. We have further addressed the effect of joystick usage in a control experiment that compared the neural correlates of error expectation when participants either used the joystick as in the calibration phase of the two experiments or just monitored the robot trajectories while someone else controlled the joystick (see subsection “Effects of joystick usage on ErrP”).

### ErrP-decoder performance

Figure 3c reports classification accuracy of the ErrP decoder in four different conditions. Supplementary Figs. 4 and 5 report individual confusion matrices of the four conditions for the first and the second experiments, respectively. We collected 61 ± 18 correct and 11 ± 4 erroneous trials in the first, and 88 ± 42 correct and 18 ± 15 erroneous trials in the second experiment per subject. On average across all subjects, 84 ± 8%, 71 ± 9%, 83 ± 11%, and 70 ± 13% (mean ± std) of classification accuracy was obtained for Offline-Timelock, Offline-Continuous, Online-Timelock and Online-Continuous decodings, respectively. Offline performance measures were determined with data from the calibration phase performing a 10-fold cross validation, while Online decodings report classification accuracy achieved during the adaptation phase when performing the classification analysis either in a time-lock or in a continuous manner. As expected, Timelock reached the highest performance for Offline and Online conditions as it was evaluated on the time-locked windows used to build the ErrP decoder. This accuracy substantially decreases during the continuous evaluation of the decoder along the robot trajectory in the adaptation phase. Nevertheless, Online-Continuous did not further decrease with respect to Offline-Continuous. Furthermore, as illustrated in Fig. 3d, despite this lower online performance, the mean latency across all 13 subjects in decoding ErrPs with respect to the moment when subjects released the joystick was 0.76 ± 0.15 s. This value was very close to the latency of the decoding with the Time-Lock approach, 0.5 s. The rationale for this latency of the Timelock approach was because the decoder was trained with the time window of [0.0, 0.5] s with respect to the release of the joystick. Decoding latency was similar between the two experiments, 0.78 ± 0.14 and 0.73 ± 0.18 s, respectively.

In order to assess statistical differences of the classification accuracy across the four different conditions, we performed a two-way repeated-measures ANOVA, the first factor is Offline or Online, while the second factor is Timelock or Continuous. The ANOVA revealed a significant difference between Timelock and Continuous (*F*(1, 12) = 27.1, *p* < 0.001), but not between Offline and Online (*F*(1, 12) = 0.55, *p* = 0.47), nor a significant interaction between the two factors (*F*(1, 12) = 0.004, *p* = 0.95). The subsequent post-hoc analysis with Bonferroni’s critical value correction revealed significant differences between Timelock and Continuous in both Offline and Online conditions, but not between other pairs of conditions (Fig. 3d and Table 1).

**Table 1:** Results of the post-hoc analysis between each pair of conditions, indicating the estimated difference between the corresponding two marginal means of classification performance, the standard error of the estimated difference, and the corresponding *p*-value.

Condition 1	Condition 2	Estimated difference [%]	Standard error [%]	*p*-value
Offline-Timelock	Offline-Continuous	12.7	1.8	<**0.001**
Online-Timelock	Online-Continuous	12.9	3.3	**0.002**
Offline-Timelock	Online-Timelock	1.0	1.6	0.55
Offline-Continuous	Online-Continuous	1.27	1.8	0.49

Statistically significant *p*-values are in bold

We further analyzed the relationship between the ErrP classification performance and the release rate during the adaptation phase of the two experiments by performing Pearson’s correlation coefficient analysis for the conditions Online-Continuous (*r*(13) = −0.655, *p*(13) = 0.015) and Online-Timelock (*r*(13) = −0.770, *p*(13) = 0.002) (Supplementary Fig. 6). As expected, subjects with lower ErrP classification performance had a higher release rate of the joystick due to inaccurate weighting of the robot trajectories.

### Learning the desired parameters with inverse reinforcement learning

In the first experiment, we investigated the robot trajectories with or without the release, and the error rate of the calibration phase to understand when and how often participants released the mouse over the course of the calibration phase. As shown in Fig. 3e, participants released the joystick when the robot was moving along a lower trajectory, and so passing closer to the obstacle, compared to the trajectory in which participants did not release the joystick. The rationale for this behavior is that these lower trajectories elicited error expectation. Upon the release, we observed an elbow shape in the trajectory to avoid the obstacle without collision. The number of collisions was 4 ± 1 per subject during the first batch with random modulation parameters, i.e., before the modulation parameters were generated by IRL. Additionally, we analyzed the release rate during the calibration phase to examine if there was a trend in the subjects’ behavior over time. Figure 3f illustrates the error rate for each 10% interval of the calibration phase; a Pearson’s correlation revealed no trend of the release rate across subjects during the course of calibration (*r*(130) = 0.037, *p*(130) = 0.67), endorsing no effect of habituation on releasing the joystick.

As shown in the previous subsection, the performance of the ErrP decoder varies among the subjects, and its accuracy decreases during online operation. If the output of the ErrP decoder were used directly to control the assistive robotic arm, its low performance would result in inconsistent and unreliable trajectories. Here, we present a method for dealing with this issue and increasing the efficiency of the robot. Figure 2 illustrates the control approach. To learn the preferred trajectories for each subject, we associate each demonstrated trajectory with a weight coming from the posterior probability of the ErrP decoder (*W* = 1 − *PostProb*(*ErrP*)). Then, we employ an IRL method with these demonstrations to converge to the desired trajectory.

The first step of the IRL consists of an update of the *reward* function. The reward function is an implicit model of the subject’s costs function. In our implementation, IRL expresses the reward function as a weighted linear combination of Gaussian kernels, which are radial basis functions, centered on the obstacle. Each kernel has a different width. The superposition of the kernels delineates the preferred regions around the obstacle. As learning proceeds, the width for each kernel changes and the region may be enlarged or shrunk to reflect the subject’s preference.

**Fig. 4:**
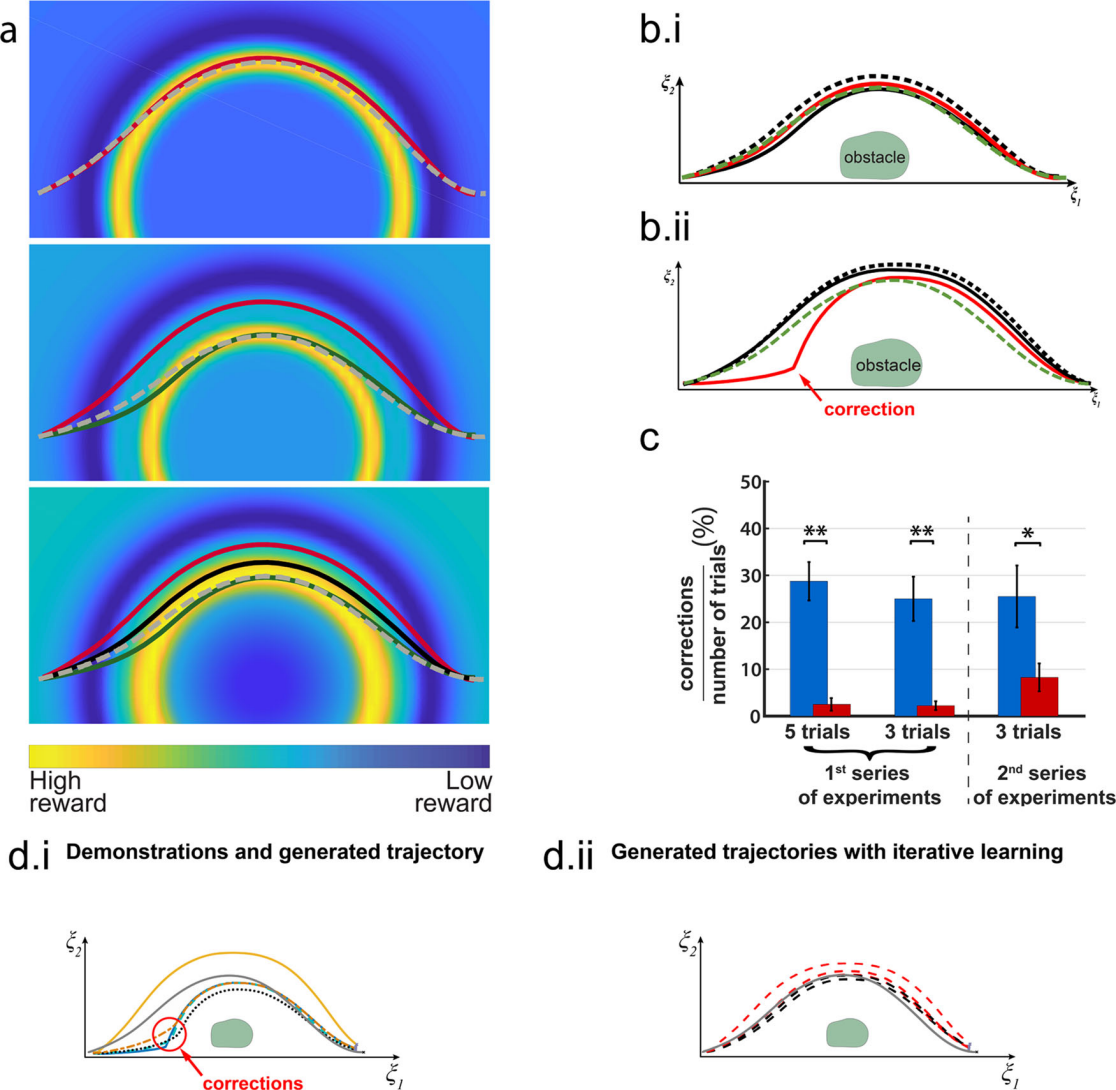
Evolution of the reward function and of the robot’s trajectories during learning. **a** Representative example of the evolution of the reward function when increasing the number of demonstrations. The warmer color corresponds to the areas with high reward whilst the cooler color to areas of low reward. Since the Gaussian kernels are centered on the position of the obstacle, the area with a high reward becomes circular. The gray dashed lines correspond to the trajectory generated by the IRL method and the red, green, and black line correspond to the successive adaptation trajectories. **b** Two examples of addressing the uncertainty of the ErrP decoder. The IRL infers the green dashed line on the basis of the two black (continuous and dashed) and the red lines. In both examples, the output of the ErrP decoder for red trajectories is inconsistent with respect to the other trajectories. i. Although the red trajectory is very similar to the black trajectories with large weights, the ErrP decoder assigns a small weight to the red one. ii. Despite the red trajectory corresponds to a correction from the subject, the ErrP decoder assigns it a large weight. Nevertheless, the inconsistencies of the ErrP decoder have no effect on the inferred trajectory; it remains closer to the dark trajectories than to the red trajectories. **c** Average ratio and standard error between the number of corrected trials over the number of overall trials before (i.e., during the calibration phase) and during the adaptation phase of the IRL method for the first and second experiments. Subjects corrected the robot motion significantly less times during the adaptation of IRL for both the first and second experiment: *p* < 0.001 and *p* = 0.0443, respectively. **d** Representative example of the effect of the ErrP-decoder output. The generated trajectories are between to the demonstrations with higher weights (smaller output from the ErrP-decoder) and the demonstrations with a lower weight. Left graph: the demonstrations and their weights that have been used to generate the preferred trajectory with our IRL approach. Right graph: the generated trajectories during iterative learning.

Figure 4a illustrates the evolution of the reward function after three consecutive rounds of IRL adaptation for one of the subjects. As mentioned, updates are influenced by the probability of having decoded an ErrP. The lower the probability, the higher importance is given to the trajectory. This is visible by looking at the first two learning rounds (first two graphs in Fig. 4a). The introduction of a demonstration with a significantly higher weight than the previous one moves the area with high reward (yellow ring) closer to this demonstration. As more demonstrations are introduced, the size of the area with high reward increases (third graph of Fig. 4a), covering the demonstrations of high weight and rejecting the demonstration with lower weight. Thus, the generated trajectory (gray dashed line in Fig. 4a) is closer to the trajectories of high weight.

In practice, the ErrP decoder may assign quite different weights to similar trajectories as depicted in Fig. 4b-i. Although the trajectories (black and red lines) are similar, the ErrP decoder assigns a significantly smaller weight to the red trajectory with respect to the two black trajectories (0.91 and 0.81 to the black trajectories and 0.35 to the red trajectory). Nevertheless, the weighted IRL tolerates this inconsistency and generates a trajectory (i.e., green dashed line) in between the trajectories the system considers to be correct. Figure 4d further illustrates how our IRL module handles the variability of the ErrP decoder, which not always assign the correct weights to the trajectories. The ErrP-decoder assigned small weights to 4 out of the 5 first demonstrations (Fig. 4d-i). As a result, the new generated trajectory lies in-between these four demonstrations and the uncorrected demonstration to which the ErrP decoder assigned a weight of 1. For the next 5 robot trajectories (Fig. 4d-ii), the subject applied no correction although the ErrP-decoder assigned different weights to each of the trajectories, indicating differences in the subjective evaluation of their quality. Hence, the IRL-based learning scheme manages to produce a trajectory closer to the preferred trajectory for this subject. Furthermore, although no corrections were applied by the subject, 2 out of the 5 robot trajectories in Fig. 4d-ii had weights less than 0.4, likely indicating cases of false positives. Similar to the previous case, the weighted IRL tolerates the inconsistency of the ErrP-decoder.

Besides the above case of false positives, the variability of the EEG signals could also make the ErrP decoder generate false negatives; i.e., the decoder fails to detect the ErrP associated to a robot trajectory which the subject did correct. Figure 4b-ii shows such a case where the subject applied a correction to the red trajectory that leads to a sharp change of direction. However, the ErrP decoder falsely assigned a large weight (0.84%) to this trajectory, in the same range as the black continuous trajectory for which the subject did not apply any correction. Still, the erroneously classified trajectory had only a minor influence on the new trajectory generated by the IRL module, which remains in close proximity to the other two (correct) trajectories. Thus, the weighted IRL approach exhibits a high tolerance to inconsistencies of the ErrP decoder.

The approach is not only robust to the natural variability and sub-optimal performance of the ErrP decoder, but it also converges rather quickly. Indeed, once the modulation parameters are generated by our learning scheme (i.e., after the first 3 or 5 trajectories, depending on the experiment), the subject did not need to correct the robot motion in the large majority of the ensuing trajectories, as shown in Fig. 4c. In both experiments, the number of corrections during adaptation is significantly lower than before IRL initialization (i.e., initial trajectories during the calibration phase generated with random modulation parameters). Specifically, a two-sample t-test on the ratio of the number of corrections over the overall trials before and during adaptation returned *p* < 0.001 in the first experiment (60 corrections out of 224 trials before adaptation and 9 out of 336 during iterative adaptation, over all eight subjects), regardless of the number of trials used for IRL initialization, and *p* = 0.0443 in the second experiment (41 corrections out of 192 trials before adaptation and 24 out of 320 during adaptation, over all five subjects). Supplementary Fig. 7 presents the percentage of corrections of each subject. Interestingly, in the first experiment, the subjects corrected the robot trajectories 2.2 ± 1.0% and 2.5 ± 1.3% of the adaptation trials when using 3 and 5 demonstrations for IRL initialization, respectively. A two-sample t-test between the number of corrections when the number of trials used for IRL initialization was 3 or 5 showed no significant differences (*p* = 0.87). This indicates that 3 demonstrations are efficient for identifying the subject’s preferred trajectories. Furthermore, no significant differences were noticed between the frequency of corrections and the stage of the adaptation phase; the corrections were not concentrated on specific sets of trials (one-sample *t*-test over the frequency of correction occurrences over all subjects, *p* = 0.78 for the 3 demonstrations and *p* = 0.65 for the 5 demonstrations).

We further evaluated this assumption of fast IRL convergence in the second experiment, where the subjects were asked to interact with the robot to perform more complex pick-and-place tasks. The subjects corrected 8.25 ± 3% of the adaptation trials, 3.75 times more than in the first experiment (*p* = 0.04). The increase in the number of corrections was expected due to the increase on the task complexity, since this protocol not only involved avoiding obstacles, but also picking and moving objects to multiple targets. In addition, the subject’s viewpoint to the robot motion affected the number of corrections. Although no significant differences were noticed on the frequency of corrections among the different robot motions (*p* = 0.58, one-way ANOVA), the subjects corrected on average 65.9% less the robot motion when the motion direction was perpendicular to the subject’s field of view (perpendicular to the sagittal plane) than sideways. No significant differences were noticed between the frequency of corrections and the stage of the adaptation phase; the corrections were not concentrated on specific sets of trials (*p* = 0.58, an one-sample *t*-test over the frequency of correction occurrences over all subjects). Moreover, all the subjects drove the robot to all of the four potential targets, for more details see Supplementary Fig. 8.

**Fig. 5:**
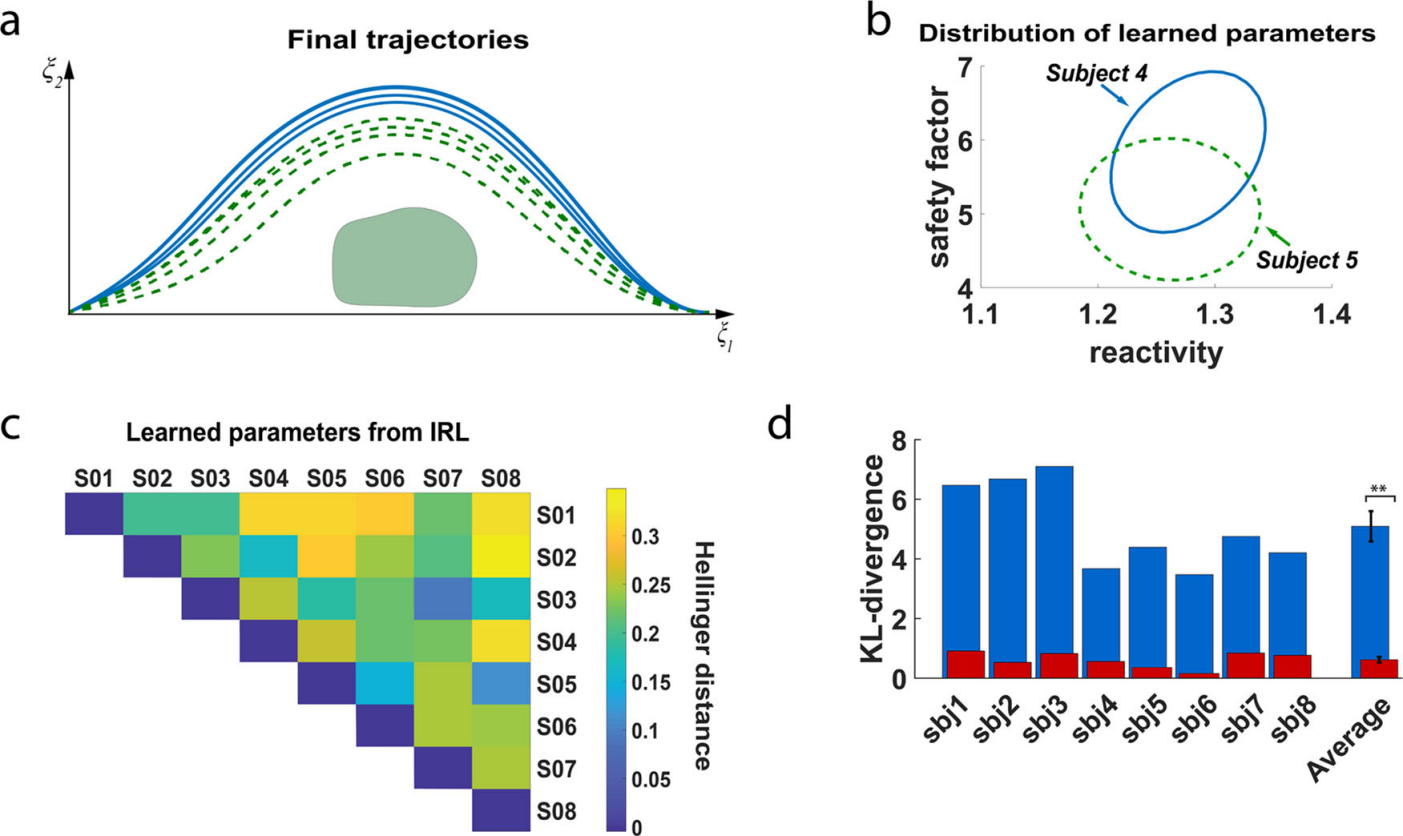
Final parameters and robot’s trajectories after learning. **a** Example of preferred trajectories for two subjects in experiment 1 (Subjects 4 and 5). Subject 5 prefers trajectories closer to the obstacle. **b** The distributions of the learned modulation parameters for Subjects 4 and 5. The preferences of the two subjects are depicted on the different distributions of the learned parameters. The distribution of the learned parameters for Subject 4 occupies a higher region than the one of Subject 5. **c** Map of Hellinger distances among the distributions of the learned modulation parameters between all the subjects of experiment 1. **d** Comparison of the KL-divergence (mean ± std) between the distributions of the modulation parameters learned by the IRL method against the parameters corrected and the parameters accepted by the subject during the calibration phase (400 trajectories of which ~25% were perceived as erroneous by subjects) used for calibration of the ErrP decoder. **p < 10^−3^.

Furthermore, it is worth noting that our approach enables the customization of robot trajectories according to the subject’s preference. Figure 5a presents the final trajectories of two subjects for the four sets of adaptation trials (i.e., the learned trajectories at the end of each evaluation) in experiment 1 when using 3 initial demonstrations. The trajectories of subject 5 pass closer to the obstacle than those of subject 4 who prefers a more conservative robot behavior. Also, and importantly, the learned trajectories for each subject are consistent. Customization to individual preferences is also depicted on the distribution of the learned DS-modulation parameters (Fig. 5b) for the two subjects, which are different. This is also the case across all subjects as illustrated in Fig. 5c. Supplementary Figs. 9 and 10 provide more details on the learned parameters for all subjects in experiment 1. Although, given the small number of parameters, there is an overlap across subjects, the values of the parameters still result in different personalized trajectories. Moreover, as shown in Fig. 5d, the convergence to individual parameters is not arbitrary, but reflects the actual behavior of the subjects during the calibration phase. Taking as a reference the subjects’ behavior during the calibration phase (400 trajectories of which 25% were perceived as erroneous by subjects) used for calibration of the individual ErrP decoders, the KL-divergence is significantly lower between the distributions of the modulation parameters learned by the IRL method and the parameters accepted by the subject during the calibration phase than between the distributions of learned parameters and the parameters corrected by the subject during the calibration phase (two-sample *t*-test, *p* < 10^−3^). This indicates that the learned parameters correspond indeed to a subset of the parameters the subject considers acceptable.

### Effects of joystick usage on ErrP

Although the neural correlate of error expectation when participants released the joystick have the properties of an ErrP, the question arises as to whether error expectation without the use of the joystick would have the same neural correlate. To answer this question, and rule out that the neural correlate of error expectation is not elicited by the interaction with the joystick or overlapping components, we performed a control experiment in a setup identical to the calibration phase of the two experiments, where the robot arm moved from one side of the table to the other while avoiding an obstacle in the middle. In this control experiment participants either used the joystick as before (condition “with-joystick”) or monitored the robot trajectories while an operator utilized the joystick, never releasing it even if the robot arm collided with the object (condition “without-joystick”).

Supplementary Fig. 11 shows the grand-averaged signals of the two classes, i.e., erroneous and correct, in the two conditions, i.e., with-joystick and without-joystick. We collected 232 ± 39 correct and 64 ± 34 erroneous trials in the with-joystick condition, as well as 272 ± 16 correct and 31 ± 15 erroneous trials in the without-joystick condition. We observed a reduced number of erroneous trials in the without-joystick condition as participants indicated their subjective preferences retrospectively, i.e., after completing a trial. The number of collisions between the end-effector and the obstacle was 3 ± 2 times in the with-joystick condition, whereas it was 6 ± 2 times in the without-joystick condition. To confirm whether these time-locked neural responses are significantly different due to the motor action of releasing the joystick, we performed a Wilcoxon’s signed-rank test for each time sample of the signals between the with- and without-joystick conditions for each class, followed by a Benjamini–Hochberg false discovery rate correction^30,31^. The statistical analysis revealed no significant difference between the two conditions for both the erroneous and correct classes. Furthermore, we computed the Pearson’s correlation coefficient of the two conditions within the time window of [−0.1 0.4] s, independently for each class (erroneous: *r*(256) = 0.869, *p*(256) < 0.001; correct: *r*(256) = 0.006, *p*(256) = 0.93). These results confirm that the neural correlate of error expectation is elicited by the perception of an erroneous trajectory that leads to a collision and not by the interaction with the joystick or multiple overlapping components.

Supplementary Table 2 presents an overall summary of the experiments and their results. Supplementary Video 1 presents an overview of our methods and results together with our robotic implementation.

## Discussion

We have described and experimentally validated a novel approach for assistive robotic manipulators that people with residual motor capabilities, although lacking fine control, can easily operate and rapidly train to perform desired behaviors. Our approach combines inverse reinforcement learning (IRL) techniques and brain–computer interfaces (BCI) that decode error-related potentials (ErrP), enabling the robotic system to infer a reward function from the subject’s ErrP that leads to individual preferred control policies without requiring the participant to make it explicit. Such a combination avoids the need to collect optimal demonstrations, something that people suffering from severe motor disabilities can hardly do. Instead, the intelligent robotic manipulator automatically generates trajectories that are weighted by the output of the ErrP decoder, indicating whether or not the user considers them appropriate. These weighted trajectories are continuously fed to the IRL module to achieve seamless adaptation to the user’s preferences.

Results not only show the feasibility of our ErrP-IRL approach and the rapid incremental learning of desired robot motion from a short number of demonstrations (Fig. 4), but also that our approach enables the customization of robot trajectories according to the subject’s individual preferences (Fig. 5). Critically, the learned parameters reflect the actual behavior of the subjects during the calibration phase. Furthermore, since our approach requires a small number of training samples, it scales very well, and exhibits good generalization capabilities, to more complex tasks like in our second experiment where the systems learns the subject’s preferred trajectories in 8 different conditions for pick-and-place tasks while avoiding other objects on the table. Another key property of our approach is its robustness to the natural variability and sub-optimal performance of the ErrP decoder when deployed online and in a continuous manner.

In contrast to other brain-controlled robot studies^4,5,7^, our approach does not require assistance from a virtual interface for controlling the robot. The increased authority on the autonomous robotic system reduces subject’s efforts to control the robot. This is advantageous over other approaches where the subject was required to continuously modulate the brain activity for controlling position^6,7,24^ and orientation^22^ of the end-effector. Correcting discrete erroneous robot actions with ErrPs and RL methods has shown great potentials in robot control^23,32,33^. Here, we extend the use of ErrPs in a number of ways: (i) they are associated not only to explicit erroneous actions, but also to error expectation; (ii) they can be decoded also during continuous robot motion; and (iii) they carry enough information to learn the subject’s preferred robot trajectories rather than only the motion direction.

In the present study, ErrP were not elicited when participants observed an explicit erroneous robot action, but by an error expectation—i.e., the moment during the robot continuous movement that the user considers will lead to an erroneous trajectory because it will not meet the participant’s preferred obstacle avoidance behavior. The results of the control experiment show that the neural correlate of error expectation is similar no matter whether subjects use a joystick to interact with the arm robot or not. This neural correlate is an event-related potential whose morphology and topography corresponds to an ErrP, as it consists of the two well-known electrophysiological negative and positive deflection around the fronto-central area of the brain, i.e., error-related negativity (ERN) and error positivity (Pe)^34,35^. ERN started deflecting even before the release of the joystick (around −0.1 s before the release, see Fig. 3a and Supplementary Fig. 3), and peaked around 0.01 s, a timing considered too early to rely on external sensory feedback^36^. Pe was observed around 0.3 s after the release, following ERN. It has been suggested that Pe may be a delayed parietal P300 associated with the perception of erroneous actions^37–39^, a hypothesis supported by the covariation of the Pe amplitude after errors in a Simon task to the P300 amplitude in response to variations in the inter-trial interval^40^.

Although ErrP has been previously exploited for teaching robots^21,23,24^, in these previous works the robot made discrete movements that facilitated the elicitation and decoding of the ErrP. Here we demonstrate the presence of ErrP even during continuous robot motion (Fig. 3), a challenge in the BCI field. Indeed, when exactly the subject considers that the robot motion is erroneous may well be an incremental decision-making process, whereby evidence is accumulated over time^41^, which varies across trajectories and subjects. To better characterize this decision, our experimental protocol asked subjects to release the joystick used for interaction in order to avoid a perceived collision. Individual ErrP decoders were trained by aligning EEG data to the time of joystick release, and then deployed in a continuous manner during the online trajectory-adaptation phase. Despite the expected decrease in performance, online continuous decoding was stable and did not further degrade with respect to the estimated offline-continuous performance with data collected during the calibration phase. Furthermore, ErrP decoding exhibited a short latency, which was very close to the latency of the optimal decoding with the Time-Lock approach (Fig. 3d).

Previous works have explored the presence of ErrPs during continuous tasks^42–48^, but in most of them, erroneous events were still generated in a discrete manner such as sudden discrepancies in the execution of commands delivered by the subjects. Omedes et al.^47^ took a step further and analyzed ErrPs arising from robot trajectories that gradually deviate from targets. Nevertheless, in their experimental protocol, the deviations happened always at the same moment along the trajectories, which facilitated the offline analysis. In our work, the robotic manipulator followed a variety of trajectories, spanning a large workspace. Deviations were variable and happened when subjects determined so, according to risks of collision. More importantly, our work is the first to have demonstrated in an online setting the possibility to decode in real-time the presence of ErrP elicited during continuous robot movements (see also recent work by Lopes-Dias et al.^48^, where ErrP were also decoded during continuous robot movements, but elicited by robot failures (stop movement) followed by a large perpendicular displacement with respect to its direction of movement).

To learn the preferred adaptation of the robot trajectories according to the subject, we integrated the output of the ErrP-BCI into an IRL scheme. Levine et al.^26,27^ show that Gaussian-Process IRL (GPIRL) is able to learn a reward function, representative of the task, even with sub-optimal demonstrations. Deriving from this outcome, we associate the demonstrations with a weight, coming from the ErrP-BCI, which defines the level of optimality of the demonstrations. Different from the original formulation, we assume that we have access to this information, as conveyed by the probabilistic output of the BCI. This allows us to take advantage of both the GPIRL and the availability of naturally elicited brain signals to modulate the learning. The original GPIRL employs a modular log-likelihood which makes it vulnerable to the initial random parameters of the optimization. Although this case was rarely noticed in our experiments, a re-initialization is needed to converge to an optimum.

In our experiments, the subject directs the robot using a joystick, which is a frequent practice in controlling robot arms from individuals with motor disabilities^49–51^. However, it requires some residual distal muscle activity on the upper limb, which makes it inaccessible to subjects with severe motor impairments like paralysis (e.g., high spinal cord injury) or degenerative conditions (e.g., ALS, MS). In our experimental set-up, the joystick serves solely as a reliable indicator of the ground-truth, critical for the evaluation of our approach, and a target definition for the robot. Moreover, our learning scheme for the preferred robot trajectories avoids any input from the joystick, and depends only on the output of the ErrP decoder and the robot demonstrations. Hence, the joystick interface could be replaced with another interface, e.g., a eye-tracker, for target selection, without modifying our control approach. There is evidence that gaze and visual guidance can be used for the selection of robot actions^52–54^. Since the subject is not required to constantly modulate the brain signals in our approach, using an additional module for target selection based on vision should not increase subject’s fatigue to unacceptable levels.

Generating robot trajectories using dynamical systems (DS) provides the robotic system with the flexibility to rapidly modify the robot trajectories for avoiding the obstacles, whilst guaranteeing the system’s convergence to the goal. Since the trajectory modification depends on two parameters, we exploit this characteristic for relating the output of the ErrP-decoder to the desired robot trajectories. In our work, we modulate an originally linear DS based on these two parameters in order to avoid convex obstacles. Since this method guarantees the stability of the system, our approach could be further expanded to the modulation of non-linear dynamical systems^12^ and to non-convex obstacles^13^. However, the obstacle avoidance method modifies the trajectories of the end-effector of the robotic manipulator, whilst the overall configuration of the robotic manipulator depends on an inverse-kinematics solver (e.g., ik-solver). As the ik-solver is agnostic of the obstacle’s position and shape, the solution from the ik-solver might result in the collision of the obstacle with another robot-link. In this work, we address this issue by letting the robotic manipulator move above the obstacles, assuming that all the objects and obstacles are placed on a plane. The introduction of the obstacles’ characteristics into the ik-solver decrease the possibility of collision^55,56^. The incorporation of these approaches to our system could enable the obstacle avoidance in the joint-level of the robotic manipulation without removing the benefits of trajectory generation of the robot’s end-effector from a DS.

Another limitation of our approach is that, in our current implementation, obstacles were static and located in pre-defined positions. To bring this work closer to a real-world application, the robot should be able to detect the targets and obstacles automatically, possibly through vision, exploiting recent advances in object detection^57^ and localization^58^. Once the moving obstacle detected, our control algorithm can be used again as it can safely avoid moving obstacles^13^. As object recognition may not be perfect, subjects may need to correct further the robot’s behavior, this time with IRL acting on the perception. Such an extension is part of our future work.

The position of the robotic manipulator with respect to the subject is an important component of an assistive robotic system. In our experiments, the subjects were outside the workspace of the robot manipulator due to safety reasons. This set-up is, however, different from a typical set-up of a robotic manipulator being attached to a wheelchair of an individual with disabilities. Having a robot manipulator close to the user may have a positive or a negative effect on the user’s perception of erroneous robot motion: because of the proximity errors might be perceived easier and with a larger valence or, on the contrary, create distractions and loss of focus due to the operation of the robot (e.g., noise coming from the robot). Moving forward to a real-life application, it would be important to test the proposed learning scheme on these real-life conditions.

Looking towards the future, we aim to further design and develop teaching and control methods for increasing the dexterity of external prostheses whilst facilitating the interaction for the subject. Future assistive robotic manipulators should involve autonomous grasping for an increased grasp stability in a larger variety of objects. The ultimate goal will be to introduce a seamless human-machine coordination, capable for performing complex tasks in real-world environments. This would, however, dramatically increase the number of variables of the control system that can be tuned. While learning in our experiments was quick and required few initial demonstrations, increasing the number of variables may lead to ambiguities during learning and far more training time and examples. We will require novel machine learning and BCI paradigms to prune the search space of robot’s parameters and rapidly identify those that subjects consider relevant and require to be updated.

## Methods

### Experimental protocol

Thirteen able-bodied subjects, within 20 to 34 y/o (24.8 ± 3.2), with no prior knowledge of neurological disorder participated in the study. Experimental protocols were approved by the local ethics commission (Cantonal Ethical Committee, PB_2017-00295) and all experiments were carried out in accordance with the approved guidelines. Informed consent was obtained from all participants that volunteered to perform the experiments.

We conducted two experiments with different tasks. Eight subjects participated in the first experiment and five subjects did in the second experiment. In these experiments, the subject always directed the end-effector of the robot arm with the joystick, while the robot autonomously performed an obstacle avoidance of an object placed in the middle of the trajectory, as described in the subsection “Dynamical system and obstacle avoidance”. Each experiment consisted of two phases; namely, the decoder-calibration phase and the trajectory-adaptation phase.

In the decoder-calibration phase of the two experiments, the subject made the robot go left or right by deflecting the joystick roughly in that direction. The robot moved as long as the joystick was pressed while attempting to avoid an obstacle. The two DS-modulation parameters that determine the robot trajectory were chosen at random (sampled from homogeneous distributions) for each trial. This design was chosen to ensure that we would generate a diversity of trajectories around the object. We also allowed the robot arm to hit the object lightly. When the subject felt that the robot motion was not desirable (i.e., risk of a potential collision), s/he released the joystick. Releasing the joystick immediately modified the parameters of the DS modulation in order to increase the distance from the object and avoid a collision. A trial ended once the robot reached one of the two targets (A or B in Supplementary Fig. 1). We recorded approximately 400 trials, 295 ± 25 trials without release of the joystick (correct trials), and 110 ± 32 trials with joystick release (erroneous trials) for each subject. This recording was performed in a single session lasting 40 min. The average duration of a trial was 3.72 ± 0.04 s, and the averaged reaction time to release the joystick was 1.03 ± 0.10 s with respect to onset of the robot movement. At the end of this phase, the recorded EEG signals were used to train an ErrP decoder. We observed collisions between the end-effector of the robot and the obstacle for 8 subjects, 4 ± 1 times per subject, during the calibration phase.

Once the ErrP decoder was trained, we continued to the trajectory-adaptation phase, where the IRL method was combined with the output of the ErrP decoder to learn the preferable DS-modulation parameters. To ensure that the experimental conditions remained comparable across the calibration and adaptation phases, subjects were instructed to interact with the robot in the same way in both phases. Specifically, the subject directed the robot to go left or right with the joystick, whilst the robot was attempting to perform an obstacle avoidance. The subjects were allowed to release the joystick and correct the robot trajectories, if they perceived a potential collision (note that IRL will not necessarily lead to learning preferred DS parameters similar to this automatic, safety behavior of the controller upon joystick release (i.e., DS parameters that increase the distance to objects), since it accepts the robot trajectories and not the corrected DS-modulation parameters.) As IRL requires a batch of demonstrations to initiate the adaptation process, we evaluated two batch-sizes: 3 or 5 demonstrations. For this initial batch of demonstrations, the DS-modulation parameters were chosen randomly. Afterward, the DS-modulation parameters for the following trials were generated from our weighted-IRL method.

In this second phase, we evaluated (1) the output of the ErrP decoder and (2) the performance of the control approach with the utilization of IRL. Hence, the trajectory-adaptation phase corresponded to our testing phase. The metric of performance for the learning of the preferred trajectories was the number of trials in which the subjects modified the DS modulation (by releasing the joystick) after the IRL method was initially trained with the first batch of demonstrations.

In the first experiment, the trajectory-adaptation phase consisted of eight sets of ten trials. In four out of the eight sets of trials, the batch size for the IRL was 5 demonstrations and for the remaining four sets of trials the batch size was 3.

In the trajectory-adaptation of the second experiment, we increased the complexity of the robot motion bringing it closer to a real application. Specifically, the subject controlled the robot to pick and place objects from/to four positions, releasing the joystick in case of error expectation. We placed different obstacles in between the target positions, letting the robot perform obstacle avoidance autonomously. The subject selected the target position with the joystick and to grasp or release the object by pressing the joystick downwards. Since this experimental setup involved more targets, the IRL method learned trajectories for eight conditions; one for each set of targets times the factor grasping/not-grasping the object for each target. Specifically, IRL learned two preferred trajectories for each target position; one when the gripper was grasping the object and one when the gripper was open (i.e., without grasping an object). Figure 1c shows the location of targets and obstacles and the functionality of the joystick. The subject freely directed the robot to move towards one of the four targets and to grasp or release the object. Once a batch of 3 demonstrations for each condition were acquired, using random DS-modulation parameters, the IRL method was trained and produced the preferable modulation for each condition. Afterward, the subject performed 40 additional trials, where the latest five demonstrations were used iteratively to retrain the IRL method for the chosen condition. Subjects repeated the trajectory-adaptation phase of the second experiment twice.

### Dynamical system and obstacle avoidance

The robot trajectories are generated from a linear first-order autonomous dynamical system (DS):

1 $$\dot{\xi }=f(\xi )$$

where *f*: *ℜ*^*d*^ → *ℜ*^*d*^ is a linear continuous and continuously differentiable function with a single equilibrium point ${\dot{\xi }}^{\star }=f({\xi }^{\star })=0$. The equilibrium point represents the target’s position. The use of autonomous dynamical systems enables the generation of the robot trajectories in real-time utilizing only the current robot’s state (i.e., the position of the end-effector) and the target’s position. Therefore, once the location of the target is defined, the end-effector of the robot arm moves towards the target following linear trajectories.

To avoid obstacles, the initial linear dynamical system can be modulated locally around the object^12,13^. In our implementation, we model the obstacles’ boundary with a sphere centered on the object ξ^o^ and with diameter *r*^o^, which can be computed rapidly at run time. By applying this modulation to the dynamical system, the Eq. (1) becomes:

2 $$\dot{\xi }=M(\xi ;{\xi }^{{{{{{\mathrm{o}}}}}}},{{{{{{{{\bf{r}}}}}}}}}^{{{{{{\mathrm{o}}}}}}},\rho ,\eta )f(\xi )$$

The modulation factor *M*(ξ; ξ^o^, **r**^o^, *ρ*, *η*) deforms locally the original dynamics *f* such that the end-effector of the robot moves around the object. The avoidance trajectories can be customized by two parameters; the safety factor (*η*) and the reactivity (*ρ*). The parameter *η* corresponds to the safety margin from the obstacle whilst *ρ* effects the magnitude of the modulation.

In our approach, we define a priori the parameters of the obstacle and let the subject customize the DS-modulation parameters *ρ* and *η* exploiting the ErrPs elicited from their brain activity.

### Inverse reinforcement learning

Let us say that a task is defined by continuous states ${{{{{{{\boldsymbol{x}}}}}}}}={({{{{{{{{\boldsymbol{x}}}}}}}}}_{1},...,{{{{{{{{\boldsymbol{x}}}}}}}}}_{T})}^{T}$ and continuous actions ${{{{{{{\boldsymbol{u}}}}}}}}={({{{{{{{{\boldsymbol{u}}}}}}}}}_{1},...,{{{{{{{{\boldsymbol{u}}}}}}}}}_{T})}^{T}$, such that the next state is a result of the previous state and the corresponding action:

3 $${{{{{{{\mathcal{F}}}}}}}}({{{{{{{{\boldsymbol{x}}}}}}}}}_{t-1},{{{{{{{{\boldsymbol{u}}}}}}}}}_{t})={{{{{{{{\boldsymbol{x}}}}}}}}}_{t}$$

The actions of each state are defined by a policy *π* as a result of a reward function *r*(***x***_*t*_, ***u***_*t*_). inverse reinforcement learning (IRL) utilizes the observations of optimal behaviors from experts in order to learn a reward function that describes the human actions^59^. The observations correspond to sample paths of an agent that follows an underline policy *π*^⋆^. In this paper, we employ IRL for learning the most preferable robot trajectories according to the subject.

In our approach, the agent corresponds to the end-effector of the robot while the observations are the demonstrations of the robot motion, i.e., the trajectories of the end-effector. Let us define the state as the position of the end-effector ξ and its velocity $\dot{\xi }$ as the action **u**. Due to the non-linear trajectories of the demonstrations, we employ a Gaussian process (GP) as the reward function for mapping the feature values to rewards, similar to the approach of Levine et al.^26,27^. Specifically, the GP covariance **K**_*ij*_ is a variant of the radial basis function kernel:

4 $$k({f}^{i},{f}^{j})=\beta exp\left(-\frac{1}{2}\mathop{\sum}\limits_{k}{\lambda }_{k}[{({f}^{i}-{f}^{j})}^{2}+{1}_{i\ne j}{\sigma }^{2}]\right)$$

where *σ*^2^ is the regularized noise. We introduce a set of features ${{{{{{{\bf{F}}}}}}}}={[{{{{{{{{\bf{f}}}}}}}}}^{1}...{{{{{{{{\bf{f}}}}}}}}}^{n}]}^{T}$ to the GP, which are induced from the points of the demonstrated trajectories, and learn the output noiseless trajectory **y** together with the kernel parameters *λ* and *β*. To induce the features, we use an elliptical base function (EBF) kernel, so that the corresponding features **f**_*i*_ of a point are:

5 $${{{{{{{{\bf{f}}}}}}}}}^{i}=exp\left(\!\!-\frac{\zeta }{2}{({{{{{{{\boldsymbol{\xi }}}}}}}}-{{{{{{{\boldsymbol{\mu }}}}}}}})}^{T}{{\Lambda }}({{{{{{{\boldsymbol{\xi }}}}}}}}-{{{{{{{\boldsymbol{\mu }}}}}}}})\right)$$

where ζ and Λ correspond to hyper parameters. The center points of the EBF kernel are the center of the obstacle. We put three kernels at each obstacles, with different width ζ and covariance matrix Λ. More kernels lead to more computation time and three kernels is sufficient in our experiment.

In order to find the preferred trajectories, we need to maximize the following GP likelihood:

6 $${{{{{{{\mathcal{L}}}}}}}}={{{{{\mathrm{log}}}}}}{{{{{{{\mathcal{P}}}}}}}}({{{{{{{\bf{y}}}}}}}},\lambda ,\beta | {{{{{{{\bf{F}}}}}}}})=-\frac{1}{2}{{{{{{{{\bf{y}}}}}}}}}^{T}{{{{{{{{\bf{K}}}}}}}}}^{-1}{{{{{{{\bf{y}}}}}}}}-\frac{1}{2}{{{{{\mathrm{log}}}}}}| {{{{{{{\bf{K}}}}}}}}| +{{{{{\mathrm{log}}}}}}{{{{{{{\mathcal{P}}}}}}}}(\lambda ,\beta | {{{{{{{\bf{F}}}}}}}})$$

where *P*(*λ*, *β*∣**F**) corresponds to hyper parameters prior that guarantees the sparsity of *λ* and prevents degeneracies that occur when **y** → 0. Following the work of Levine et al.^27^, we select this prior to be:

7 $${{{{{\mathrm{log}}}}}}{{{{{{{\mathcal{P}}}}}}}}(\lambda ,\beta | {{{{{{{\bf{F}}}}}}}})=-\frac{1}{2}{{{{{\mathrm{tr}}}}}}({{{{{{{{\bf{K}}}}}}}}}^{-2})-\mathop{\sum}\limits_{k}{{{{{\mathrm{log}}}}}}({\lambda }_{k}+1)$$

Once the likelihood is optimized, we can use the reward to retrieve the expert’s policy. The reward at a feature point **f**(**x**_*t*_, **u**_*t*_) is given by the GP posterior mean:

8 $$r({{{{{{{{\bf{x}}}}}}}}}_{t},{{{{{{{{\bf{u}}}}}}}}}_{t})={{{{{{{{\bf{K}}}}}}}}}_{\star ,{{{{{{{\bf{y}}}}}}}}}^{T}{{{{{{{{\bf{K}}}}}}}}}^{-1}{{{{{{{\bf{y}}}}}}}}$$

where **K**_⋆,**y**_ is the covariance between **f**(**x**_*t*_, **u**_*t*_) and the inducing points. In the case of multiple demonstrations, we maximize the sum of the accumulated likelihood:

9 $${{{{{{{{\mathcal{L}}}}}}}}}_{N}=\mathop{\sum}\limits_{n}{{{{{{{{\mathcal{L}}}}}}}}}_{i}$$

with *n* being the number of demonstrations introduced to the method.

A fundamental assumption of IRL stands on the optimality of the provided demonstrations. However, this is rarely the case, especially in control methods that require input from neurophysiological signals due to their low signal-to-noise ratio. In this work, we address this uncertainty by assigning a weight *w*_*i*_ to the provided demonstration in relation to the brain activity of the subject. Thus, the optimized likelihood becomes:

10 $${{{{{{{{\mathcal{L}}}}}}}}}_{N}=\mathop{\sum}\limits_{n}{w}_{i}{{{{{{{{\mathcal{L}}}}}}}}}_{i}$$

where *w*_*i*_ = 1 − PP^ErrP^, with PP^ErrP^ being the posterior probability that outputs the ErrP decoder.

Once we learn the reward function from IRL, we compute the corresponding modulation of the DS. Since the modulation is described by a pair of parameters (*ρ*, *η*), we employ the basic gradient-free gradient descent, using non-linear simplex, to compute the modulation parameters which give the closest reward to the one computed by IRL. The gradients of *ρ* and *η* for each step *i* (${g}_{{\rho }_{i}}$ and ${g}_{{\eta }_{i}}$ accordingly) are given from the formulas below:

11 $${g}_{{\rho }_{i}}=\frac{R(\rho ,\eta )-R(\rho +\epsilon ,\eta )}{\epsilon }$$

12 $${g}_{{\eta }_{i}}=\frac{R(\rho ,\eta )-R(\rho ,\eta +\varepsilon )}{\epsilon }$$

where *ϵ* and *R* correspond to the learning step (selected to be 10^−3^) and the learned reward accordingly. In our experiments, GP-IRL required 10–15 iterations for producing a trajectory, regardless of the number of demonstrations introduced (3 or 5). The computational time required for the generation of a new set of DS-modulation parameters was between 10 and 40 s; 13 ± 2.4 and 32 ± 5.8 s when the initial batch consisted of 3 demonstrations and 5 demonstrations, respectively.

We further investigate the differences among three types of modulation parameters across subjects: (1) the parameters of the trajectories corrected by the subjects during the calibration phase used to calibrate the individual ErrP decoders, considered as erroneous parameters; (2) the parameters of the trajectories that the subjects did not correct during the calibration phase, considered as correct parameters; and (3) the parameters learned by the IRL method, or learned parameters. For each subject, we model the parameters of each of the above three clusters with Gaussian distributions. We use the Kullback–Leibler divergence (KL-divergence)^60^ for exploring the similarities among the distributions for each subject. KL-divergence is a relative measure of similarity between two distributions and, in our case, it reveals whether the learned parameters are more similar to the correct parameters than the erroneous parameters.

Additionally, we examine whether the IRL method converges to the same parameters for all the subjects or it offers a customized solution for each individual subject. To do so, we use another type of f-divergence metric, namely the squared Hellinger distance^60^. In contrast to the KL-divergence, the squared Hellinger distance is bounded with values between 0 and 1, where 0 indicates that the means of the distributions are identical, and reaches its maximum value 1 when the distributions do not overlap.

### Decoding the error-related potentials

We recorded 16 EEG and 3 electrooculogram (EOG) signals at 512 Hz via two g.USBAmps (g.tec medical technologies, Austria). EEG electrodes were located at Fz, FC3, FC1, FCz, FC2, FC4, C3, C1, Cz, C2, C4, CP3, CP1, CPz, CP2, and CP4 (10/10 international system), while the 3 EOG electrodes were placed at above the nasion and below the outer canthi of the eyes. The ground electrode was placed on the forehead (AFz) and the reference on the left earlobe. The EEG and EOG signals were notch filtered at 50 Hz to eliminate the power noise. To reduce signal contamination, participants were asked to restrict eye movements and blinks during experiments.

Before the experiment, participants underwent 90 s of recording in which they were asked to perform clockwise and counter-clockwise rolling of eyeballs, vertical and horizontal eye movements and repeated eye blinks. This data was subsequently used to compute coefficients to linearly subtract EOG artifacts from EEG signals based on the autocovariance matrix of EEG and EOG signals^61^.

We removed trials in which subjects miss-operated the joystick. Specifically, for each subject, an erroneous trial was considered anomalous and removed if it was associated with a joystick release time that deviated from the mean reaction time for that subject more than a pre-defined threshold; i.e., mean-absolute deviation (MAD) > 3.

### Time-frequency analysis

Main components of the ErrPs were identified by performing a time-frequency analysis with the Stockwell transform^62^, S-transform, on the data recorded during the decoder-calibration phase of the first and second experiments. To remove baseline drift, we firstly applied a 2nd order causal Butterworth high-pass filter with the cut-off frequency of 1 Hz. The reason for employing a causal filter is to emulate the signal processing conditions of online decoding analysis. Then, EEG signals were epoched in the time window [−0.5 1.0] s with respect to the onset of the joystick release in erroneous trials, or [1.0 2.5] s with respect to the onset of the robot movement in correct trials. S-transform *S*_*x*_(*τ*, *f*) of EEG signals *x*(*t*) is defined as follows:

13 $${W}_{x}(\tau ,d)=\int\limits_{-\infty }^{\infty }x(t)w(t-\tau ,d){{{{{\mathrm{d}}}}}}t$$

14 $${S}_{x}(\tau ,f)=\exp \left(i2\pi f\tau \right){W}_{x}(\tau ,d)$$

where *W*_*x*_(*τ*, *d*) is the wavelet transform of signal *x*(*t*) and the mother wavelet *w*(*t*, *f*) is:

15 $$w(t,f)=\frac{| f| }{\sqrt{2\pi }}\exp \left(-\frac{{t}^{2}{f}^{2}}{2}\right)\exp (-j2\pi ft){{{{{\mathrm{d}}}}}}t$$

16 $$S(\tau ,f)=\frac{| f| }{\sqrt{2\pi }}\int\limits_{-\infty }^{+\infty }\exp \left(-\frac{-{(\tau -t)}^{2}{f}^{2}}{2}\right)\exp (-2i\pi ft)x(t){{{{{\mathrm{d}}}}}}t.$$

We used two different time windows for erroneous and correct trials because, while joystick release is a natural marker for erroneous trials, no such marker exists for correct trials. For these correct trials, we opted to follow a common technique, which is to arbitrarily choose a segment of the EEG signal^18,47,48^. We employed the time window [1.5 2.0] s with respect to the onset of the robot movement, as this is the period before the robot passes around the obstacle and where the subjects should closely monitor the situation, i.e., the robot, the obstacle, and the distance between them.

The final step for the time-frequency analysis was to compute the event-related spectral perturbation (ERSP)^63^ of the erroneous trials (used as the event-related spectral, ERS) relative to the baseline *ν*:

17 $${{{{{\mathrm{ERSP}}}}}}(f,t)=10{{{{{{{\mathrm{log}}}}}}}}_{10}\left(\frac{{{{{{\mathrm{ERS}}}}}}(f,t)}{\nu (f)}\right)$$

where the baseline is the averaged power of correct trials in the time window [1.5 2.0] s with respect to the onset of robot movement, which corresponds to the time window of correct trials in subsequent classification analysis. The rationale for the baseline time window was to avoid the presence of visually-evoked event-related potentials at the beginning of the trajectory, and keep the consistency with the time window used to create the ErrP classifier (see below). The range of time and frequency was set to [−0.5 1.0] s and [1 30] Hz, respectively. Since the ErrP is known to appear over the fronto-central area^15–17^, this analysis was performed on the EEG signal at the FCz channel (Fig. 3b).

### ErrP decoding

During the IRL trajectory-adaptation phase of the first and second experiments, we decoded the presence of ErrPs online to weigh robot trajectories in a seamless fashion. To build the individually customized ErrP decoder, which infers the presence or absence of ErrPs in a continuous manner, we used the data from each participant’s individual calibration session. We first applied a 4th order causal Butterworth bandpass filter with the cut-off frequencies [1 12] Hz. Then, EEG signals were segmented into epochs: for erroneous trials, we selected the window [0.0 0.5] s with respect to the onset of joystick release, whereas for correct trials the window was [1.5 2.0] s with respect to the onset of the robot movement, which corresponds to the start of a trial. Figure 3e shows these time windows as well as the grand average of robot trajectories for erroneous and correct trials during the calibration phase of the first and second experiments.

To enhance the signal-to-noise ratio (SNR) of the EEG signals, we applied a spatial filter based on canonical correlation analysis^64–66^. This spatial filter method transforms the averaged ErrPs to a subspace containing different ERP components. Only the first three components were kept for further analysis. For every trial, we extracted three different types of features: the EEG voltage per time sample after downsampling the data to 32 Hz (48 temporal features); power spectral densities (PSD) per EEG component (15 PSD features); and features based on the Riemannian geometry, which computes a low-dimensionality manifold representation from a non-linear combination of the EEG component space^67^ (21 covariance features); thus, 84 features in total. In order to extract Riemannian features based on the spatial covariance matrix between EEG components while preserving information of temporal dynamics of the waveform, the epoch *X* was augmented with an individual template *T* representing the grand average of erroneous trials.

18 $$Z=[XT]$$

19 $${C}_{Z}=\frac{1}{s}{Z}^{T}Z=\frac{1}{s}\left(\begin{array}{ll}{X}^{T}X&{X}^{T}T\\ {T}^{T}X&{T}^{T}T\\ \end{array}\right)$$

where *s* denotes number of time samples of an epoch. The covariance between *X* and *T* allows to capture the temporal dynamics of multi-component EEG signals with respect to the template. The covariance matrix was then projected on the tangent space, which was computed based only on the calibration data set^68^. All computed features were concatenated and normalized within the range [0, 1]. Similar to our previous study^69^, we examined classification performance of different combinations of these three features, (1) temporal; (2) PSD, and (3) covariance-based features. Although we observed that temporal is the most informative feature, and temporal features alone yields similar cross-validation performance to the combined features, we decided to add PSD and covariance-based features because, based on our experience, they can slightly enhance the classification performance by taking into account the frequency power modulation and the spatial covariance of the extracted components. From this feature vector **x**, we computed the posterior probability of the presence of an ErrP, *p*(*error*∣**x**) using diagonal linear discriminant analysis:

20 $$p(error| {{{{{{{\bf{x}}}}}}}})=\frac{1}{1-{\exp }^{-({{{{{{{\bf{w}}}}}}}}^{\prime} {{{{{{{\bf{x}}}}}}}}+b)}}$$

To compute the posterior probability of the calibration data without overfitting, the aforementioned signal processing was performed in a 10-fold cross-validation manner. Training folds were used to create an ErrP decoder, while the testing fold was used to estimate continuous modulation of the posterior probability during the robot trajectory with a sliding window at 32 Hz. In order to avoid visually-evoked potentials when the robot started to move, we removed from the analysis the first 0.25 s with respect to the onset of trials; thus, the first estimated posterior probability was obtained on the time window [0.25 0.75] s from the onset of trials. This process to estimate the posterior probability continued with a sliding window approach until the robot reached to the end point (3.72 ± 0.04 s on average). Based on the computed posterior probability, 2 hyper parameters were individually optimized besides the ErrP decoder, also based only on data collected during the calibration phase: smoothing factor and decision threshold. The smoothing factor indicates the length of the time window for a moving average filter, ranging from 1 to 16 with a step of 1. The decision threshold determines whether the subject considered a trajectory erroneous during a trial, ranging from 0 to 1 with a step of 0.01. The classification algorithm infers the presence of an ErrP if the smoothed continuous posterior probability exceeds the decision threshold during the robot movement. For each pair of hyperparameters we computed the Matthew’s correlation coefficient (MCC), obtaining a 16 × 101 matrix for each testing fold. The pair of parameters with the highest MCC, averaged over the 10 testing folds, was chosen as the optimal. By using these parameters, a sigmoid function was fitted to make the decision threshold be 0.5. Once the optimal hyper parameters were determined, we used all the available calibraion data to build the ErrP decoder to be deployed online during the trajectory-adaptation phase.

To assess the classification performance of the ErrP decoder, we computed the two-class confusion matrices individually for the four different decoding modalities: Offline-Timelock, Offline-Continuous, Online-Timelock and Online-Continuous. Ground-truth of a trial was given by subjects’ behavior, release (erroneous) or no-release (correct) of the joystick. Offline performances were computed based only on the calibration data using 10-fold cross validation; while Online performances were extracted from the data of the adaptation phase in which the robot adjusted its trajectory based on ErrP-BCI output. Timelock represents the classification accuracy of the EEG epochs used to build the ErrP decoder; i.e., in the case of erroneous trials, EEG signals time locked to the onset of joystick release. Continuous represents the classification accuracy of the ErrP decoder continuously applied over the whole robot trajectory using the sliding window approach described above.

To assess the classification performance during the first and second experiments of each decoding modality, we performed a two-way repeated measures ANOVA, the first factor being Offline or Online, while the second factor was Timelock or Continuous. We collected the four different values from each subject (one value per modality). For statistical analysis of the classification performance, we combined the performance measures from the decoder-calibration and trajectory-adaptation phases of the first and second experiments as they are identical to each other. Importantly, we did not observe a significant discrepancy of the classification performance in the adaptation phases of the two experiments, i.e., Online-Continuous condition (two-sample *t*-test; *p* = 0.657).

### Effects of joystick usage on ErrP

To rule out that the neural correlate of error expectation is not elicited by the interaction with the joystick or overlapping components, we performed a control experiment in a setup identical to the calibration phase of the two previous experiments, where the robot arm moved from one side of the table to the other while avoiding an obstacle in the middle. Seven able-bodied subjects (same age distribution than in the main two experiments, 27.3 ± 1.5) took part in the control experiment; data of one of these subjects could not be analyzed due to a hardware problem that prevented synchronization of EEG recordings, joystick and robot trajectories.

We recorded the subjects’ EEG and EOG while they interacted with the arm robot in two conditions, namely, with or without the joystick. In the with-joystick condition, subjects used the joystick to make the robot arm go left or right, releasing it if they perceived the risk of a collision. In the without-joystick condition, subjects were instructed to observe the robot’s movements and pay attention to how well the robot was avoiding the glass. Only the experimenter used the joystick. Importantly, in the without-joystick condition, the experimenter never released the joystick along trajectories even if the robot arm collided with the object. Furthermore, in the without-joystick condition, subjects had to report their subjective preference on the performed robot trajectories in the range [1 10], with values [1 3] for trajectories they would have released the joystick and values above 4 if they would have kept the joystick pressed. This subjective assessment was used to classify the trials. Trials associated with the subjective report of [1 2 3] were considered erroneous trials, while trials with values [4 10] were deemed to be correct trials. In this control experiment, we recorded two runs of 150 trials for each condition while alternating the order of the conditions. The experiment always started with a run of with-joystick condition for the subjects to get accustomed to evaluate the robot trajectories.

We firstly applied a 4th order non-causal Butterworth bandpass filter with the cut-off frequencies [1 12] Hz. Then, EEG signals were segmented into epochs for each condition (with- or without-joystick): for the correct trials of both conditions, we chose the time window [1.5 2.0] s with respect to the onset of the robot movement. For the erroneous trials, the time windows depended on the condition: for the with-joystick condition, we used the time window [−0.1 0.4] s with respect to the release of the joystick (the reason for choosing an earlier time window with respect to the analysis in the two experiments is that here we use a non-causal filter, while in the latter we utilize a causal filter for online implementation, which introduces a delay); whereas for the without-joystick condition, we used the time window [−0.1 0.4] s with respect to the onset of the per-subject average reaction time to release the joystick in the with-joystick condition. Subsequently, we performed cross-correlation analysis between the per-subject average EEG for erroneous trials at FCz in the with- and without-joystick conditions to re-align the temporal waveforms of the EEG signals of the two conditions, while restricting the maximum temporal shift to be twice the standard deviation of the individual reaction time. The maximum temporal shift was chosen to cover 95% of the distribution of the actual release time in the with-joystick condition (mean ± 1.96 × std). We performed statistical tests to compare the grand-averaged signals of each class, i.e., erroneous and correct, between the two conditions based on Wilcoxon’s signed-rank test followed by the Benjamini–Hochberg false discovery rate correction. Additionally, we computed the similarity of the grand-averaged signals between the two conditions with Pearson’s correlation analysis independently for the two classes.

## Supplementary information

Supplementary Information

Description of Additional Supplementary Files

Supplementary Video 1

Supplementary Data

## Data Availability

The source data for graphs and chars are available as Supplementary Data. All physiological and empirical data is available at https://zenodo.org/record/3627015.

## Code availability

Custom code to implement obstacle avoidance movement is available at https://github.com/epfl-lasa/IRL_DS_obstacle_avoidance. Physiological analysis was performed by using MATLAB R2018b using the BioSig toolbox (http://biosig.sourceforge.net). Code for stockwell transform is available at https://www.mathworks.com/matlabcentral/fileexchange/51808-time-frequency-distribution-of-a-signal-using-s-transform-stockwell-transform. For the decoding analysis, the covariance toolbox is available at https://github.com/alexandrebarachant/covariancetoolbox.
