# Supplementary material for: Customizing skills for assistive robotic manipulators, an inverse reinforcement learning approach with error-related potentials
Source: Commun Biol. 2021 Dec 16;4:1406. doi: 10.1038/s42003-021-02891-8 (PMC8677775; doi:10.1038/s42003-021-02891-8)
Supplement: Supplementary file 1 — Supplementary Information [file 42003_2021_2891_MOESM1_ESM.pdf]

Customizing Skills for Assistive Robotic Manipulators, An Inverse Reinforcement Learning Approach with Error-Related Potentials

Iason Batzianoulis[1,*], Fumiaki Iwane[2,3,*], Shupeng Wei[1,*], Carolina Gaspar Pinto Ramos Correia[1],

Ricardo Chavarriaga[2], José del R. Millán[2,3,4,†] & Aude Billard[1,†]

Supplementary Figures

[*]These authors contributed equally to this work
[†]These authors have equally supervised this work

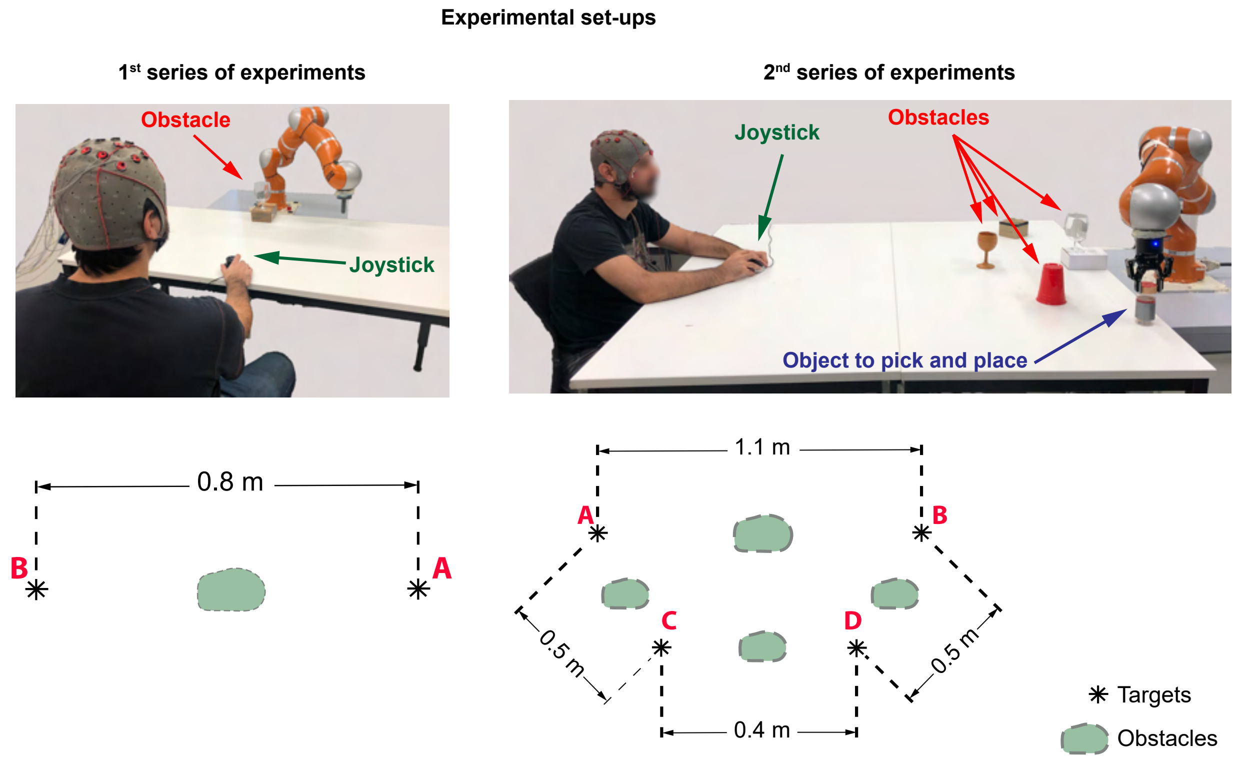

Supplemetary Figure 1: The set-up of the two series of experiments. On the bottom, a top view of the locations of the targets and obstacles.

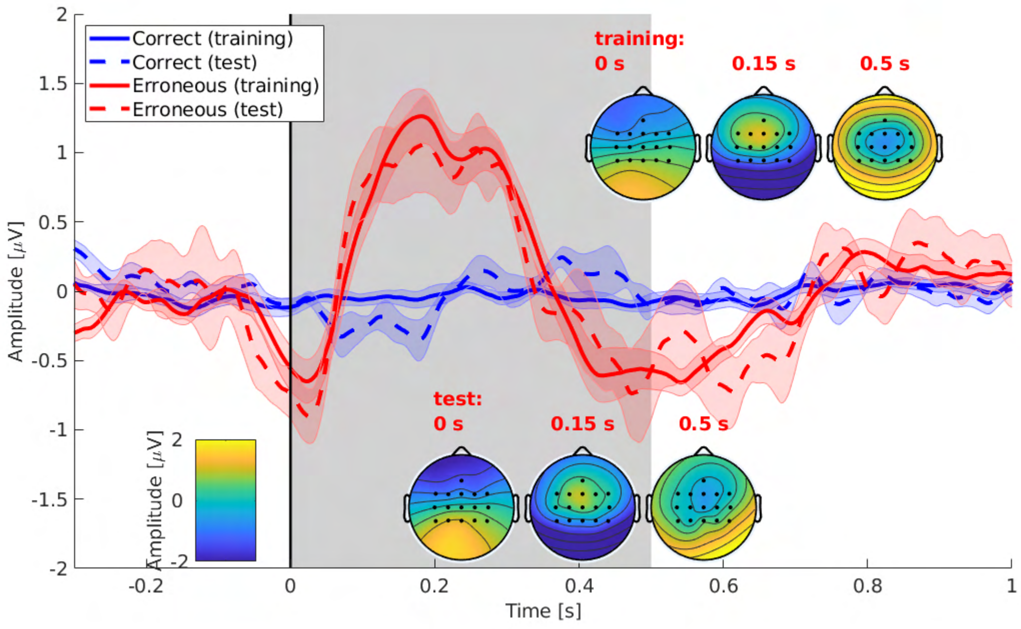

Supplemetary Figure 2: Grand-averaged signals with a causal filtering of the training data (solid lines) and test data (dash lines) of experiment 1 and 2 (N=13). We performed a Wilcoxon's signed-rank test for each time sample of the signals between the GA of the training and test datasets, followed by a Benjamini-Hochberg false discovery rate correction, which revealed no significant difference between the two. Furthermore, we computed the Pearson's correlation coefficient of the two GAs in the time window [0.0 0.5] s (erroneous: $r(256) = 0.967, p(256) < 0.001$; correct: $r(256) = 0.155, p(256) = 0.013$) that further confirmed their similarity.

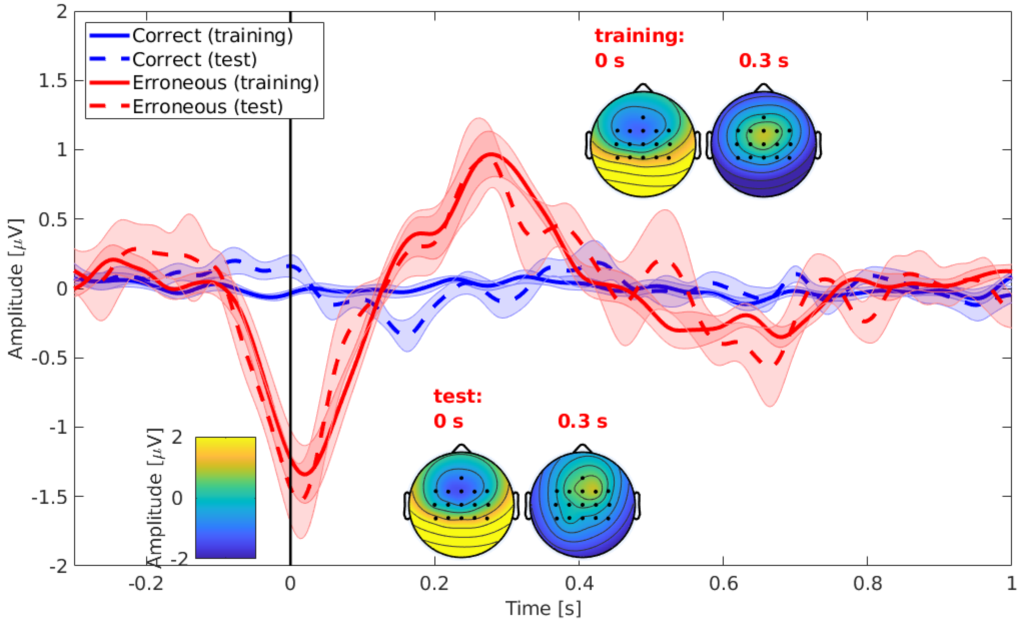

Supplemetary Figure 3: Grand average over all subjects ($N = 13$) of the EEG channel FCz with non-causal filtering (4th order bandpass butterworth filter with the cutoff frequencies of [1, 12] Hz). Inset: Topographical representation of EEG amplitude over the subjects scalp for erroneous trials at two different time points with respect to the onset (release of the joystick); i.e., 0.00 and 0.3 s.

A. S01

[%]		*Offline-Timelock*		*Offline-Continuous*		*Online-Timelock*		*Online-Continuous*	
		Predicted Label		Predicted Label		Predicted Label		Predicted Label	
		Correct	Error	Correct	Error	Correct	Error	Correct	Error
True Label	Correct	44.4	20.4	39.7	25.1	40	27.5	42.5	25
True Label	Error	14.4	20.8	13.3	21.9	20	12.5	12.5	20

B. S02

[%]		*Offline-Timelock*		*Offline-Continuous*		*Online-Timelock*		*Online-Continuous*	
		Predicted Label		Predicted Label		Predicted Label		Predicted Label	
		Correct	Error	Correct	Error	Correct	Error	Correct	Error
True Label	Correct	74.7	6.1	63.7	17.1	87.5	5	80	12.5
True Label	Error	4.9	14.3	7.9	11.3	0	7.5	7.5	0

C. S03

[%]		*Offline-Timelock*		*Offline-Continuous*		*Online-Timelock*		*Online-Continuous*	
		Predicted Label		Predicted Label		Predicted Label		Predicted Label	
		Correct	Error	Correct	Error	Correct	Error	Correct	Error
True Label	Correct	71.8	5.9	60.8	16.8	73.3	8.9	55.6	26.7
True Label	Error	2.8	19.6	6.1	16.3	6.7	11.1	10	7.8

D. S04

[%]		*Offline-Timelock*		*Offline-Continuous*		*Online-Timelock*		*Online-Continuous*	
		Predicted Label		Predicted Label		Predicted Label		Predicted Label	
		Correct	Error	Correct	Error	Correct	Error	Correct	Error
True Label	Correct	61.6	11.7	46.7	26.7	70	15	50	35
True Label	Error	11.2	15.4	10.5	16.1	8.8	6.3	5	10

E. S05

[%]		*Offline-Timelock*		*Offline-Continuous*		*Online-Timelock*		*Online-Continuous*	
		Predicted Label		Predicted Label		Predicted Label		Predicted Label	
		Correct	Error	Correct	Error	Correct	Error	Correct	Error
True Label	Correct	70.4	9.1	53.2	26.4	78.8	12.5	60	31.3
True Label	Error	6.2	14.3	6.7	13.8	3.8	5	7.5	1.3

F. S06

[%]		*Offline-Timelock*		*Offline-Continuous*		*Online-Timelock*		*Online-Continuous*	
		Predicted Label		Predicted Label		Predicted Label		Predicted Label	
		Correct	Error	Correct	Error	Correct	Error	Correct	Error
True Label	Correct	74.4	7.4	56.6	25.1	81.3	6.3	57.5	30
True Label	Error	4.6	13.7	5.6	12.7	7.5	5	7.5	5

G. S07

[%]		*Offline-Timelock*		*Offline-Continuous*		*Online-Timelock*		*Online-Continuous*	
		Predicted Label		Predicted Label		Predicted Label		Predicted Label	
		Correct	Error	Correct	Error	Correct	Error	Correct	Error
True Label	Correct	58.1	6.9	62.3	2.8	72.5	10	72.5	10
True Label	Error	8.2	26.7	21.5	13.4	3.8	13.8	15	2.5

H. S08

[%]		*Offline-Timelock*		*Offline-Continuous*		*Online-Timelock*		*Online-Continuous*	
		Predicted Label		Predicted Label		Predicted Label		Predicted Label	
		Correct	Error	Correct	Error	Correct	Error	Correct	Error
True Label	Correct	65.8	8.1	61.7	12.3	76.3	11.3	77.5	10
True Label	Error	8.1	17.9	11.5	14.5	1.3	11.3	5	7.5

Supplemetary Figure 4: Confusion matrices of the first experiment for each decoding modality and participant.

I. S09

[%] True Label	*Offline-Timelock* Predicted Label		*Offline-Continuous* Predicted Label		*Online-Timelock* Predicted Label		*Online-Continuous* Predicted Label	
	Correct	Error	Correct	Error	Correct	Error	Correct	Error
Correct	71.5	10.1	77.7	3.9	69	13.8	74.1	8.6
Error	4.9	13.5	14.2	4.1	5.2	12.1	15.5	1.7

J. S10

[%] True Label	*Offline-Timelock* Predicted Label		*Offline-Continuous* Predicted Label		*Online-Timelock* Predicted Label		*Online-Continuous* Predicted Label	
	Correct	Error	Correct	Error	Correct	Error	Correct	Error
Correct	68.7	3	69.2	2.5	94.5	3.4	95.9	2.1
Error	3.3	25	12.1	16.2	0	2.1	2.1	0

K. S11

[%] True Label	*Offline-Timelock* Predicted Label		*Offline-Continuous* Predicted Label		*Online-Timelock* Predicted Label		*Online-Continuous* Predicted Label	
	Correct	Error	Correct	Error	Correct	Error	Correct	Error
Correct	61.5	5.6	56.9	10.2	73.5	8.2	72.8	8.8
Error	5.6	27.3	14.3	18.6	11.6	6.8	14.3	4.1

L. S12

[%] True Label	*Offline-Timelock* Predicted Label		*Offline-Continuous* Predicted Label		*Online-Timelock* Predicted Label		*Online-Continuous* Predicted Label	
	Correct	Error	Correct	Error	Correct	Error	Correct	Error
Correct	50.8	10.6	51.8	9.5	67.2	14.8	50.8	31.1
Error	12.1	26.5	25.5	13.1	1.6	16.4	6.6	11.5

M. S13

[%] True Label	*Offline-Timelock* Predicted Label		*Offline-Continuous* Predicted Label		*Online-Timelock* Predicted Label		*Online-Continuous* Predicted Label	
	Correct	Error	Correct	Error	Correct	Error	Correct	Error
Correct	56.8	14.1	30.6	40.3	57.1	9.2	33.6	32.8
Error	7.1	22	7.1	22	10.9	22.7	17.6	16

Supplemetary Figure 5: Confusion matrices of the second experiment for each decoding modality and participant.

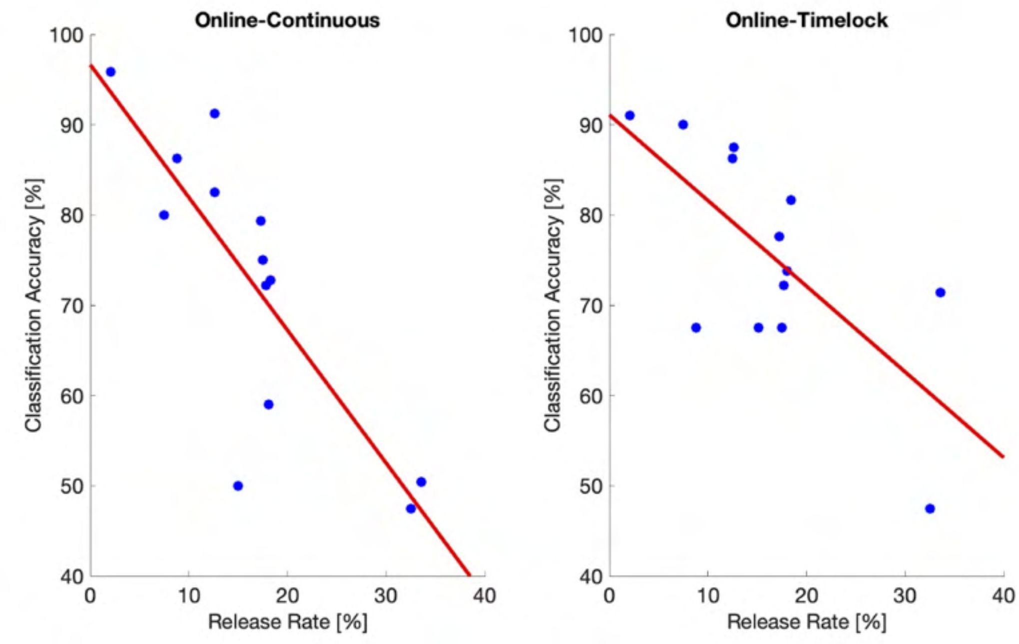

Supplemetary Figure 6: Scatter plot of the Pearson's correlation analysis between the classification performance and the release rate.

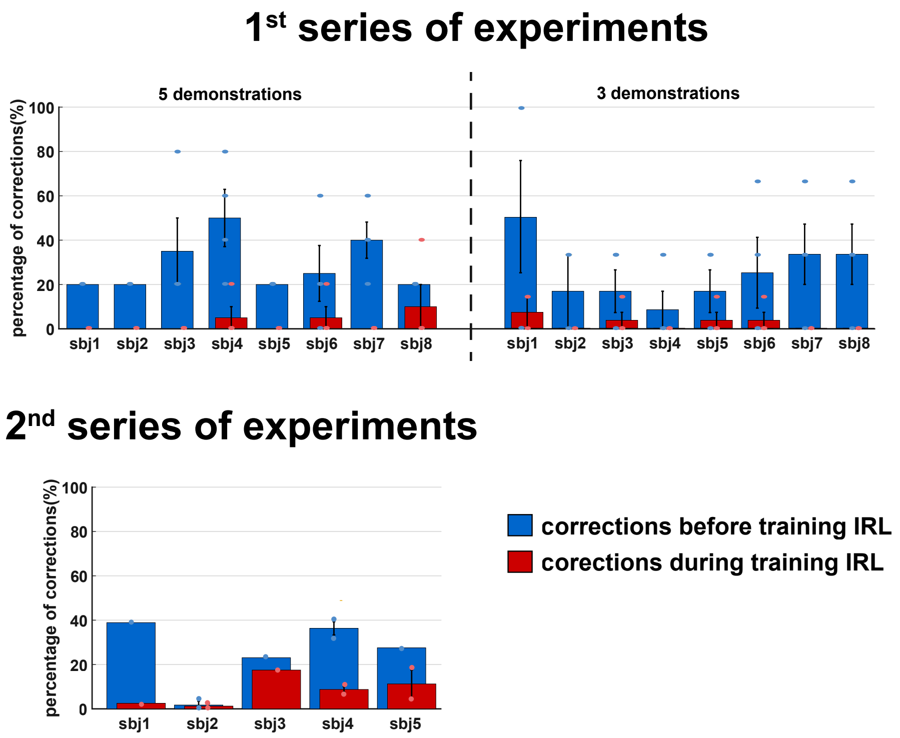

Supplemetary Figure 7: Percentage of corrections before and during learning the desired parameters with IRL. In the 1st experiment, subjects 1, 2, 3, 5 and 7 (when using 5 initial demonstrations-trials for the IRL method), and subjects 2, 4, 7 and 8 (when using 3 initial demonstrations-trials for the IRL method) did not correct the robot motion after the initial training finished. As expected, the number of corrections is larger on the 2nd experiment. However, the percentage of corrections was much lower for all the subjects, except subject 2 who made a very low number of corrections in both conditions.

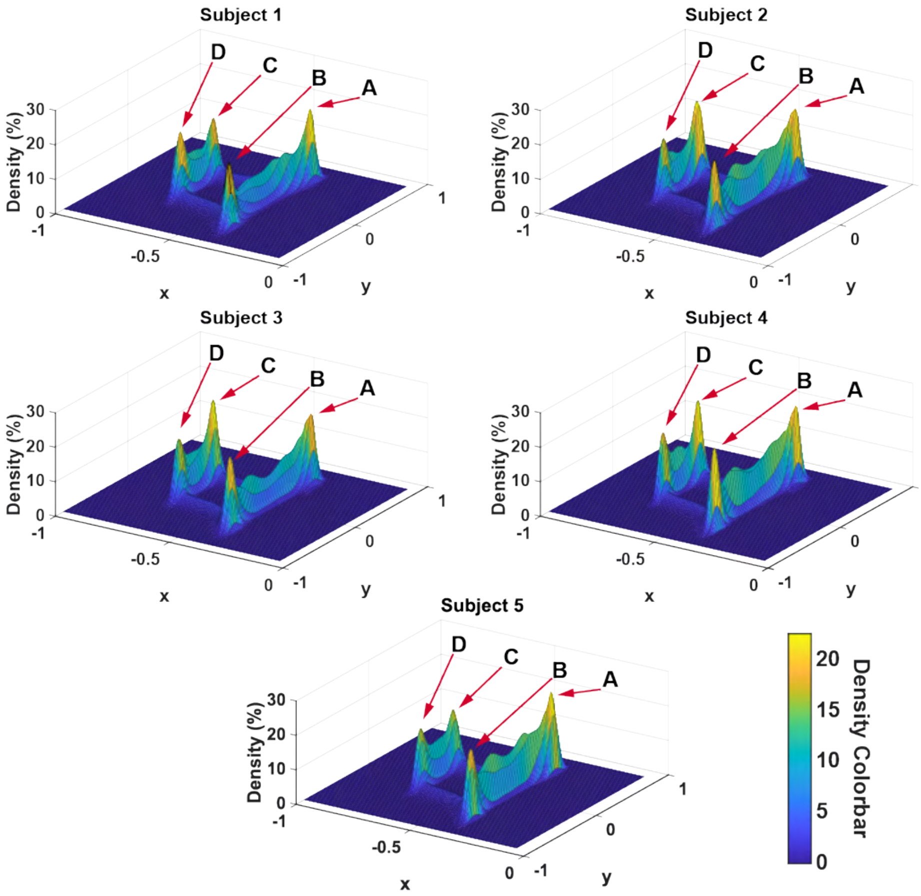

Supplemetary Figure 8: The density of the points of the robot trajectories during the testing phase of the 2nd series of experiments for all the subjects. A, B, C and D are the positions of targets. All the subjects drove the robot to all of the four potential targets.

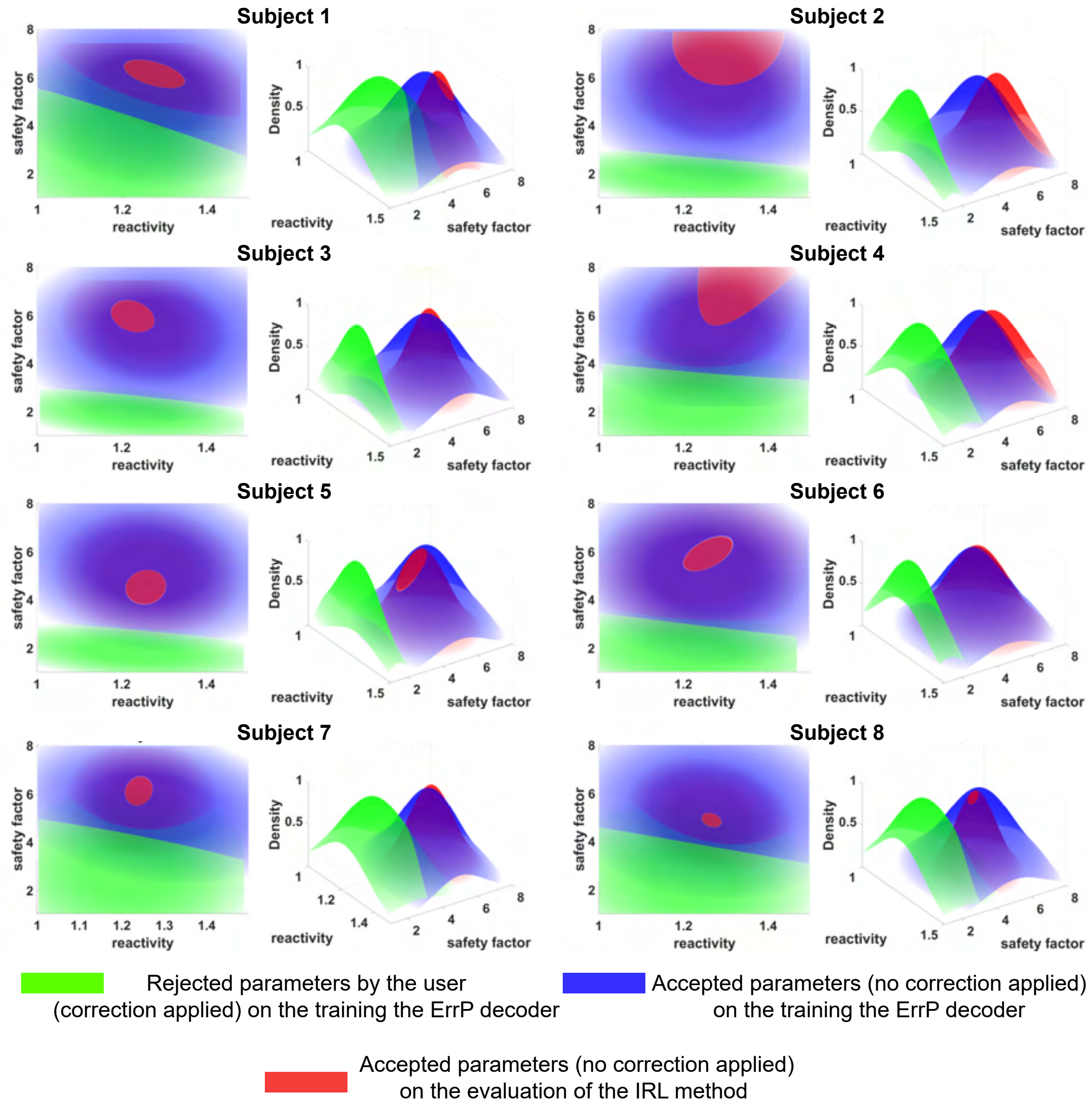

Supplemetary Figure 9: Gaussian distributions of the modulation parameters for each subject 1st series of experiments.

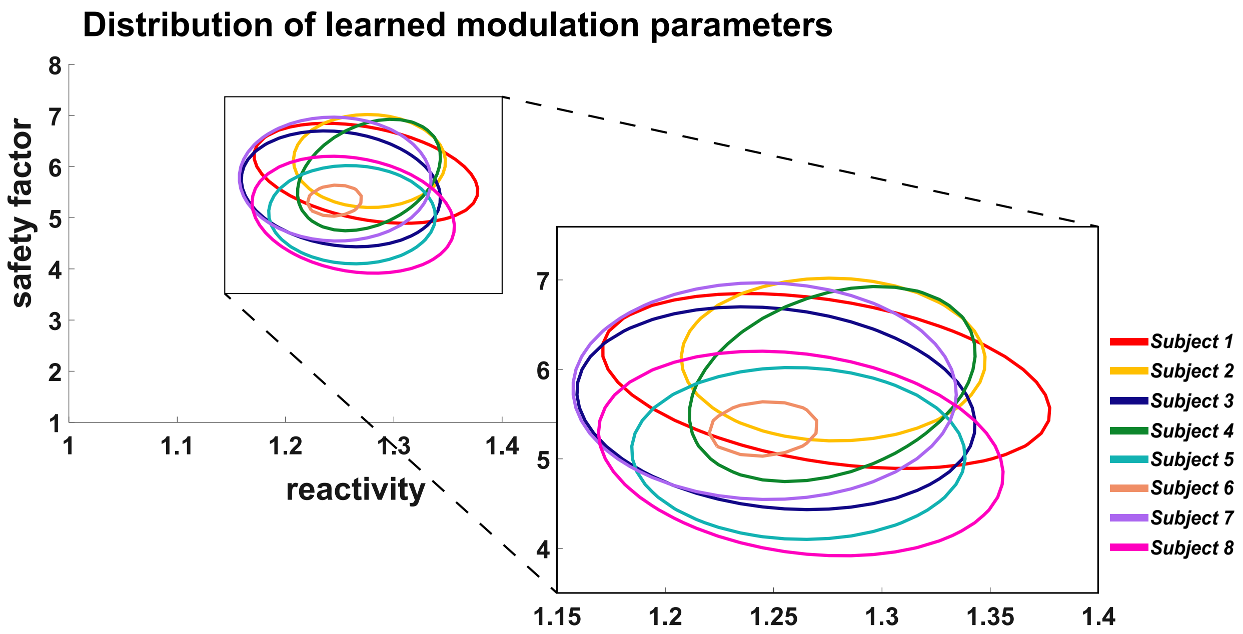

Supplemetary Figure 10: The Gaussian distributions of the learned parameters for all the subjects.

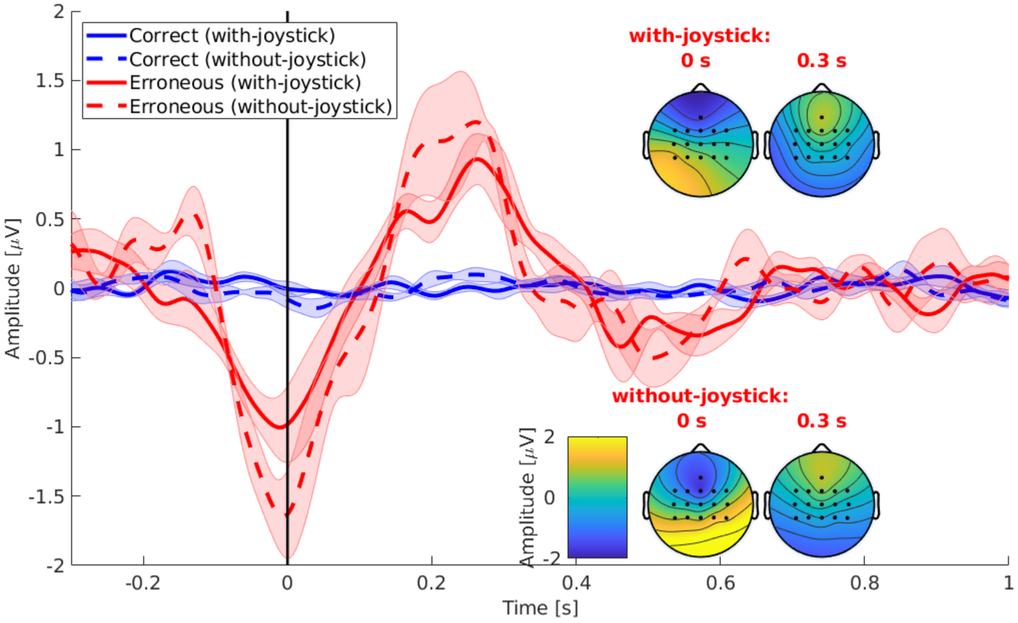

Supplemetary Figure 11: Grand-averaged signals, after re-alignment (see Section ”Methods. Effects of joystick usage on ErrP”), over all subjects in the control experiment (N=6) of the EEG channel FCz of the two classes, i.e. erroneous in red and correct in blue, in the two conditions, i.e. with-joystick in solid lines and without-joystick in dashed lines. Inset: Topographical representation of EEG amplitude over the subjects’ scalp for the grand average of erroneous trials at two different time points with respect to the onset in the two conditions; i.e. 0.00 and 0.30 s.

Supplementary Table 2: Summary of experiments and results

	1	2	3
Set-up	The robot moves towards the left or right staying on a plane perpendicular to the subjects field of view (perpendicular to the sagittal plane of the subject). One object (a plastic transparent wine glass), defined as the obstacle, is placed in the middle of the robots path. The robot follows a trajectory that attempts to avoid the obstacle depending on the modulation parameters of a dynamical system. Since the trajectory generation is agnostic to the shape of the obstacle, random modulation parameters may lead to collision with the obstacle. A joystick is used for directing and correcting the robots trajectories.	Extending the set-up to pick-and-place tasks with one object-to-grasp and 4 obstacles. A gripper, mounted on the robots end-effector, picks and places the object avoiding the obstacles in its path. A joystick is used to direct the robot, open/close the gripper and correct the robot trajectories.	Same as in experiment #1.
Goal	Combining the output of an ErrP-decoder and an Inverse Reinforcement Learning scheme for adapting the robot trajectories according to the preference of each subject.	Evaluating the ErrP-IRL scheme on pick-and place tasks.	Control experiment: ruling out that the neural correlated of error expectation is not elicited by the interaction with the joystick or overlapping components.
Conditions	ErrP-decoder calibration:<br>The subject directs the robot to move towards the left or right with a joystick. S/he keeps pressing the joystick until the end of the robot trajectory, unless there is an error expectation and releases the joystick. After joystick release, the robot increases its distance from the obstacle while moving towards the selected direction.<br>Trajectory adaptation (ErrP-IRL):<br>Two batch-sizes (3 and 5) are tested for learning the preferred robot trajectory. Robot trajectories in the initial batch are generated by random modulation parameters. Each trajectory and the associated ErrP probability are fed to the IRL module. Trajectories after the initial batch are generated by the IRL module and successively updated.	ErrP-decoder calibration:<br>Same as in experiment #1.<br>Trajectory adaptation (ErrP-IRL):<br>Initial batch-size is 3. Robot trajectories in the initial batch are generated by random modulation parameters. Each trajectory and the associated ErrP probability are fed to the IRL module. There are 4 points for the object to be picked/placed (see supplementary figure 1) and 2 motion conditions (i.e., pick the object, empty gripper; place the object, gripper holds the object). Thus, the overall number of conditions for this experiment is 8. Trajectories after the initial batch for each of the 8 conditions are generated by the IRL module and successively updated.	The subject either uses the joystick as in the ErrP calibration phase of the experiments #1 and #2 (with- joystick condition) or just monitors the robot trajectories while someone else controls the joystick (without- joystick condition). Importantly, in the without-joystick condition, the experimenter never releases the joystick along trajectories even if the robot arm collides with the object. Furthermore, in the without-joystick condition, subjects have to report their subjective preference on the performed robot trajectories in the range [1 10], with values [1 3] for trajectories they would have released the joystick and values above 4 if they would have kept the joystick pressed. This subjective assessment is used to classify the trials.
# Subjects	8	5	7
Trials	ErrP-decoder calibration:<br>4 runs containing of 100 trials each. 400 trials in total. ~25% erroneous trials.<br>Trajectory adaptation (ErrP-IRL):<br>4 runs of 10 trials for each batchsize. 80 trials in total, 48 testing trials (by removing the initial batches).	ErrP-decoder calibration:<br>Same as in experiment #1.<br>Trajectory adaptation (ErrP-IRL):<br>2 runs of 52 trials, 104 trials in total; 80 testing trials (after removing the initial batch for each condition).	2 runs of 150 trials for each condition (with- and without-joystick). 232 ± 39 correct and 64 ± 34 erroneous trials in the with-joystick condition; 272±16 correct and 31±15 erroneous trials in the without-joystick condition.
Main Results	ErrP-decoder calibration:<br>`Offline-Timelock`: 84 ± 80%;<br>`Offline-Continuous`: 70 ± 60%.<br>ErrP-decoder onine (with IRL):<br>`Online-Timelock`: 81 ± 13%;<br>`Online-Continuous`: 69 ± 10%.<br>Trajectory adaptation (ErrP-IRL):<br>- Ratio of corrections over overall trials, trajectory adaptation and initial batch (no adaptation): $p < 0.001$, two-sample t-test. Initial batch: 224 trials, 60 corrections; Trajectory adaptation: 336 trials, 9 corrections.<br>- Ratio of corrections over overall trials with adaptation, batchsize of 3 or 5: $p = 0.87$, two-sample t-test. batch-size = 3: 2.2 ± 1.0%; batch-size = 5: 2.5 ± 1.3%.<br>- One-sample t-test over the frequency of corrections across the adaptation phase per batch size: $p = 0.78$ (3); $p = 0.65$ (5).<br>- KL-divergence between the distributions of the modulation parameters learned by the IRL method against the parameters corrected and the parameters accepted by the subject during the ErrP-decoder calibration: $p < 0.001$, two-sample t-test.	ErrP-decoder calibration:<br>`Offline-Timelock`: 85 ± 70%;<br>`Offline-Continuous`: 72 ± 13%.<br>ErrP-decoder onine (with IRL):<br>`Online-Timelock`: 84 ± 70%;<br>`Online-Continuous`: 72 ± 17%.<br>Trajectory adaptation (ErrP-IRL):<br>- Ratio of corrections over overall trials, trajectory adaptation and initial batch (no adaptation): $p = 0.0443$, two-sample t-test. Initial batch: 192 trials, 41 corrections; Trajectory adaptation: 320 trials, 24 corrections.<br>- One-way ANOVA over the frequency of corrections among the 8 conditions: $p = 0.58$.<br>- One-sample t-test over the frequency of corrections across the adaptation phase: $p = 0.58$.	- No significant difference between the ErrP grand averages of the two conditions (Wilcoxons signed-rank test for each time sample of the signals between the with- and without-joystick conditions for each class, followed by a Benjamini-Hochberg false discovery rate correction).<br>- High correlation between the two grand averages of the er- roneous class ($r(256) = 0.869$, $p(256) < 0.001$, Pearsons correlation) in the time window [-0.1 0.4] s with respect to the onset of the error expectation.
