# Supplementary material for: Customizing skills for assistive robotic manipulators, an inverse reinforcement learning approach with error-related potentials
Source: Commun Biol. 2021 Dec 16;4:1406. doi: 10.1038/s42003-021-02891-8 (PMC8677775; doi:10.1038/s42003-021-02891-8)
Supplement: Supplementary file 2 — Description of Additional Supplementary Files [file 42003_2021_2891_MOESM2_ESM.pdf]

Description of Additional Supplementary Files

File name: Supplementary Video 1.
Description: Presenting the experimental protocols, methods and results of the manuscript.

File name: Supplementary Data.
Description: Source data for graphs and charts.
